# Estimating central blood pressure from aortic flow: development and assessment of algorithms

**DOI:** 10.1152/ajpheart.00241.2020

**Published:** 2020-10-16

**Authors:** Jorge Mariscal-Harana, Peter H. Charlton, Samuel Vennin, Jorge Aramburu, Mateusz Cezary Florkow, Arna van Engelen, Torben Schneider, Hubrecht de Bliek, Bram Ruijsink, Israel Valverde, Philipp Beerbaum, Heynric Grotenhuis, Marietta Charakida, Phil Chowienczyk, Spencer J. Sherwin, Jordi Alastruey

**Affiliations:** 1 Department of Biomedical Engineering, School of Biomedical Engineering and Imaging Sciences, King’s College London, King’s Health Partners, London, United Kingdom; 2 Department of Clinical Pharmacology, King’s College London, King’s Health Partners, London, United Kingdom; 3 TECNUN Escuela de Ingenieros, Universidad de Navarra, Donostia-San Sebastián, Spain; 4 Philips Research, Cambridge, United Kingdom; 5 Philips Healthcare UK, Philips Centre, Guildford Business Park, Guildford, Surrey, United Kingdom; 6 HSDP Clinical Platforms, Philips Healthcare, Eindhoven, The Netherlands; 7 Department of Cardiology, University Medical Centre Utrecht, Utrecht, The Netherlands; 8 Cardiovascular Pathophysiology, Institute of Biomedicine of Seville, University Hospital of Virgen del Rocío, University of Seville, CIBERCV, CSIC, Seville, Spain; 9 Department of Pediatric Cardiology and Intensive Care, Hannover Medical School, Hannover, Germany; 10 Department of Pediatric Cardiology, University Medical Center Utrecht/Wilhelmina Children’s Hospital, Utrecht, The Netherlands; 11 Department of Aeronautics, South Kensington Campus, Imperial College London, London, United Kingdom; 12 Institute of Personalized Medicine, Sechenov University, Moscow, Russia

**Keywords:** blood flow models, central blood pressure, magnetic resonance imaging, ultrasound, virtual subjects

## Abstract

Central blood pressure (cBP) is a highly prognostic cardiovascular (CV) risk factor whose accurate, invasive assessment is costly and carries risks to patients. We developed and assessed novel algorithms for estimating cBP from noninvasive aortic hemodynamic data and a peripheral blood pressure measurement. These algorithms were created using three blood flow models: the two- and three-element Windkessel (0-D) models and a one-dimensional (1-D) model of the thoracic aorta. We tested new and existing methods for estimating CV parameters (left ventricular ejection time, outflow BP, arterial resistance and compliance, pulse wave velocity, and characteristic impedance) required for the cBP algorithms, using virtual (simulated) subjects (*n* = 19,646) for which reference CV parameters were known exactly. We then tested the cBP algorithms using virtual subjects (*n* = 4,064), for which reference cBP were available free of measurement error, and clinical datasets containing invasive (*n* = 10) and noninvasive (*n* = 171) reference cBP waves across a wide range of CV conditions. The 1-D algorithm outperformed the 0-D algorithms when the aortic vascular geometry was available, achieving central systolic blood pressure (cSBP) errors≤2.1 ± 9.7mmHg and root-mean-square errors (RMSEs)≤6.4 ± 2.8mmHg against invasive reference cBP waves (*n* = 10). When the aortic geometry was unavailable, the three-element 0-D algorithm achieved cSBP errors ≤ 6.0 ± 4.7mmHg and RMSEs ≤ 5.9 ± 2.4mmHg against noninvasive reference cBP waves (*n* = 171), outperforming the two-element 0-D algorithm. All CV parameters were estimated with mean percentage errors ≤ 8.2%, except for the aortic characteristic impedance (≤13.4%), which affected the three-element 0-D algorithm’s performance. The freely available algorithms developed in this work enable fast and accurate calculation of the cBP wave and CV parameters in datasets containing noninvasive ultrasound or magnetic resonance imaging data.

**New & Noteworthy:**

First, our proposed methods for CV parameter estimation and a comprehensive set of methods from the literature were tested using in silico and clinical datasets. Second, optimized algorithms for estimating cBP from aortic flow were developed and tested for a wide range of cBP morphologies, including catheter cBP data. Third, a dataset of simulated cBP waves was created using a three-element Windkessel model. Fourth, the Windkessel model dataset and optimized algorithms are freely available.

## ■ Introduction

Recent clinical studies have shown that central (aortic) blood pressure (cBP) is a better cardiovascular risk indicator than brachial blood pressure (bBP) (1–4), since cBP is more representative of the load exerted on major organs (1, 5). Regardless of gender or disease, cBPs in subjects with similar brachial systolic blood pressure (SBP) may differ by up to 33mmHg, resulting in “a significant overlap of central SBP scores between brachial SBP risk groups” (6). Furthermore, bBP can be misleading in healthy young adults due to central-brachial pulse pressure (PP) amplification of up to 30mmHg (7). The most direct method to measure cBP is cardiac catheterization, which is costly and carries risks to patients (e.g., blood clot formation and embolization) due to its invasive nature, even when performed in specialized centers (2). Consequently, there is great value in developing methods for estimating cBP noninvasively that are less risky and more suitable for frequent use.

A potential approach is to use a computational model of the circulation to estimate cBP from noninvasive measurements of aortic flow and peripheral blood pressure (BP) (8). Aortic flow can be measured using magnetic resonance imaging (MRI) or ultrasound (US). Peripheral systolic and diastolic BP can be easily measured using a brachial cuff, whereas a peripheral BP wave can be measured using, for example, applanation tonometry. MRI can also measure vascular geometry, which can be used to further refine the model—the importance of aortic geometry was proposed by Westerhof et al. (9). Consequently, computational models could be personalized to estimate cBP in cardiac MRI and US settings. Moreover, these imaging modalities are the gold standard when assessing cardiac anatomy (cardiac magnetic resonance and echocardiography). Combining the information they provide with the knowledge of cBP could enable the noninvasive derivation of pressure-volume loops and myocardial wall stress, two major indicators of cardiac performance. Although previous studies have used reduced-order models to estimate cBP noninvasively, they either did not use patient-specific MRI aortic geometry (10) or did not validate their cBP estimates against invasive cBP measurements or compare the performance of several algorithms (8, 11–14).

**Figure 1:**
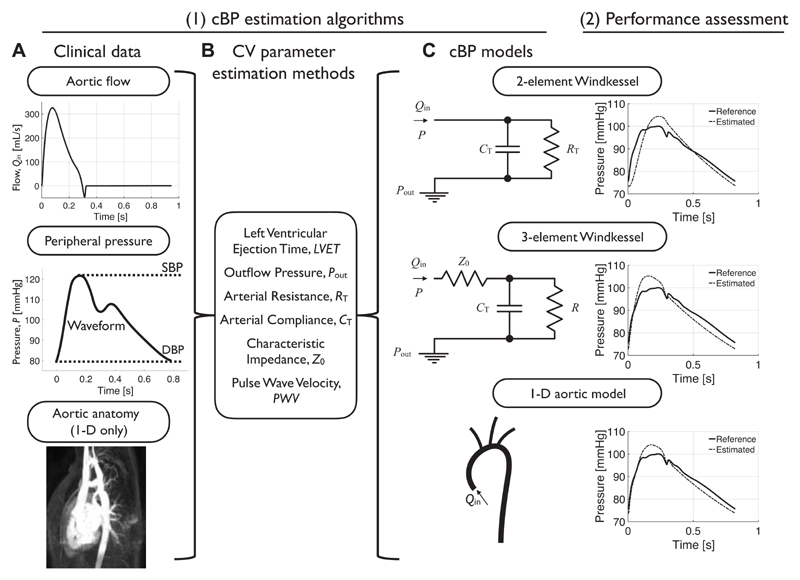
Study methodology. *1*) Central blood pressure (cBP) estimation algorithms consisted of three steps. *A*: clinical data acquisition and preprocessing: blood flow measured at the ascending and descending [one-dimensional (1-D) algorithm only] aorta; peripheral blood pressure (BP) measurement; and aortic anatomy (1-D algorithm only). *B*: cardiovascular (CV) parameters estimated from clinical data. *C*: these parameters, along with the noninvasive clinical data, were used as inputs to one of three cBP models. *2*) Algorithm performance was assessed by comparing cBP estimates provided by each model to reference values.

The aim of this study was to develop and assess three novel algorithms of increasing complexity for estimating the cBP wave from aortic flow, using noninvasive, patient-specific data from the thoracic aorta (Fig. 1). Each algorithm used a different blood flow model: the two-element (15) and three-element (16) zero-dimensional (0-D) Windkessel models and a one-dimensional (1-D) model of the thoracic aorta (11). The first step in each algorithm was to estimate cardiovascular (CV) parameters from noninvasive hemodynamic data measured in the thoracic aorta and a peripheral BP measurement. These CV parameters were left ventricular ejection time (LVET), outflow vascular BP (*P*_out_), total arterial resistance (*R*_T_) and compliance (*C*_T_), aortic pulse wave velocity (PWV), and characteristic impedance (*Z*_0_). The second step was to use these parameters as inputs to one of the three blood flow models to estimate a patient-specific cBP waveform. In this study, we assessed the performance of the CV parameter estimation methods and cBP algorithms against reference data, including invasive cBP measurements.

## ■ Methods

### Datasets

The CV parameter estimation methods and cBP algorithms were initially developed and tested using two datasets of virtual subjects. The cBP algorithms were then assessed using three clinical datasets. The characteristics of each dataset are shown in Table 1.

#### Clinical datasets

The first clinical dataset, called the aortic coarctation dataset, contains data acquired from 10 patients with aortic coarctation (17). The St Thomas’ Hospital Research Ethics Committee approved this prospective study, and informed consent was obtained from all patients (ethics reference number R&D REC 08/H0804/134). Inclusion criteria comprised native or residual aortic coarctation. Exclusion criteria were the presence of stented aortic coarctation or aortic dissection. Data were acquired in a hybrid magnetic resonance/X-ray suite guidance system. A 1.5-T MRI scanner (Philips Intera, Philips, Best, The Netherlands) was used to obtain a breath-hold three-dimensional contrast-enhanced angiography of the thoracic aorta (used to obtain aortic geometry measurements) and free-breathing two-dimensional phase contrast flow velocity through-plane scans at the ascending and upper-descending aortas (used to obtain flows at both locations). Invasive BP data were measured using X-ray-guided cardiac catheterization (Philips BV Pulsera). Measurements were taken simultaneously at the ascending and descending aortas, immediately after the flow acquisition, using multipurpose catheters (angiographic catheter 4F with carbon dioxide-filled balloon).

The second and third clinical datasets, called the normotensive and hypertensive datasets, were obtained from (18): *1*) 13 normotensive healthy volunteers at baseline and after the administration of different doses of four inotropic and vasoactive drugs (dobutamine, norepinephrine, phentolamine, and nitroglycerin) and *2*) 158 subjects assessed for hypertension (including those found to be normotensive). Both datasets were approved by the London-Westminster Research Ethics Committee, and written informed consent was obtained. Aortic flow was obtained by Doppler sonography, and peripheral BP measurements were obtained by carotid applanation tonometry. Reference cBP measurements were acquired using the SphygmoCor system (AtCor Medical, Sydney, Australia), which uses a transfer function to calculate cBP from carotid BP measured noninvasively by applanation tonometry (1,19).

**Figure 2:**
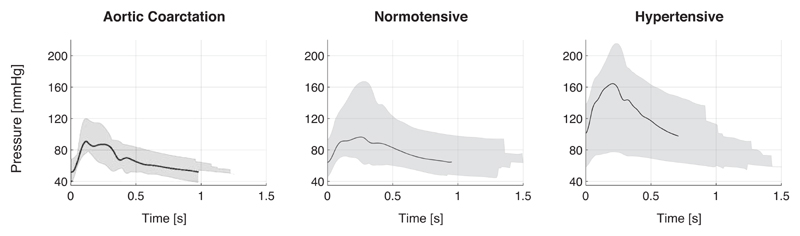
Clinical central blood pressure (cBP) wave morphologies: (*left*) aortic coarctation dataset (obtained invasively), (*middle*) normotensive (noninvasive) dataset, and (*right*) hypertensive (noninvasive) dataset. Black lines illustrate a random patient’s cBP waveform. Shaded regions represent the range of cBP waves within each dataset.

The range of cBP waves contained within each clinical dataset is shown in Fig. 2.

#### Datasets of virtual subjects

**Figure 3:**
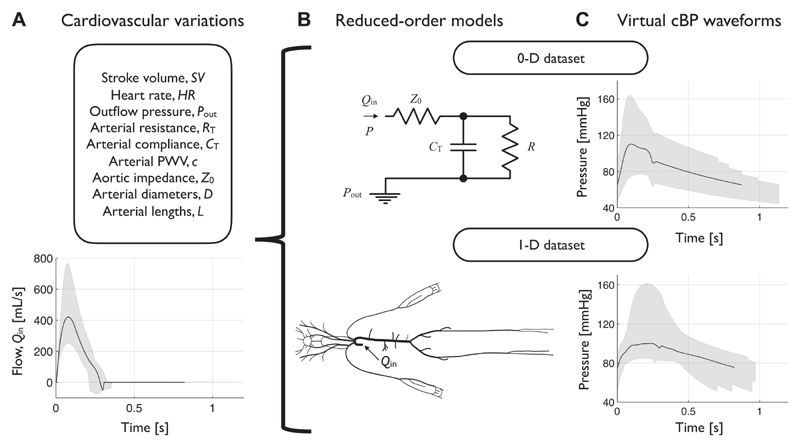
Generating datasets of virtual subjects. *A, top*: A range of values for each cardiovascular (CV) parameter was obtained from the clinical literature for healthy individuals (see Table A1). *A, bottom*: the thick line illustrates the flow wave corresponding to the baseline values of stroke volume (SV) and heart rate (HR), and the shaded region represents the range of flow waves corresponding to all SV and HR variations. B: two reduced-order models were used to generate central blood pressure (cBP) waves. C: cBP waves generated by each model: black lines illustrate the cBP wave corresponding to the baseline set of parameter variations, and shaded regions represent the range of cBP waves within each dataset.

Two datasets of BP and flow waves measured in virtual subjects were created by simulating arterial hemodynamics using 0-D and 1-D computational models, respectively (Fig. 3). A new 0-D dataset, whose reference CV parameter values were known precisely, was used to initially test existing CV parameter estimation methods and develop new ones. An existing 1-D dataset was used to further test and refine these methods and the cBP estimation algorithms, as it is based on a more physiological model of the arterial circulation (20).

The 0-D dataset was created using a three-element Windkessel model (see, *Central Blood Pressure Estimation Algorithms*). Each virtual subject’s cBP wave was simulated using an aortic flow wave generated by the *AorticFlowWave* script (21) based on prescribed values of heart rate (HR) and stroke volume (SV) in combination with prescribed values of *R*_T_, *C*_T_, *Z*_0_, and *P*_out_. CV parameters were selected to create a dataset of cBP waves representative of a sample of healthy adults. To do so, *1*) mean (μ) and standard deviation (σ) values of each parameter in healthy adults were identified from the literature (see appendix A); 2) five values for each parameter were calculated as μ, μ ± 0.5σ, and μ ± σ; and 3) a virtual subject was created using each of the 15,625 combinations of CV parameters.

The 1-D dataset was created by using a 1-D blood flow model in the aorta and larger arteries of the head and limbs. The CV properties of 25–75-yr-olds were identified through a comprehensive literature review. Pressure, flow velocity, and luminal area waves were simulated in the aorta and other common measurement sites of 4,374 virtual subjects and were verified by comparison against clinical data [see (20) for full details].

We removed nonphysiological data from further analysis, based on limits derived from the hypertensive and normotensive datasets (see Table 1). Maximum limits of central systolic BP (cSBP) and central pulse pressure (cPP) were obtained from the hypertensive dataset. Minimum limits of central diastolic BP (cDBP) and cPP were obtained from the normotensive dataset. Consequently, we excluded subjects with cSBP > 220 mmHg, cDBP < 44 mmHg, and cPP < 18 mmHg or >109 mmHg. Forty-three subjects were excluded from the 0-D dataset; 310 subjects were excluded from the 1-D dataset.

### Cardiovascular Parameter Estimation Methods

The following CV parameters were required as inputs to at least one of the cBP estimation algorithms: LVET, *P*_out_, *R*_T_, *C*_T_, *Z*_0_, and aortic pulse wave velocity (PWV). A comprehensive literature review of CV parameter estimation methods was performed. The methods listed in Table 2 and described in appendix B were implemented and assessed in this study. To be included, they had to satisfy at least one of the following inclusion criteria: they were reported as the optimal method (22–26), their performance was similar to that of the optimal method (23, 24, 26–28), they were the only reported method (15, 29–47), or their performance had not been sufficiently assessed due to their novelty (32, 35, 37). In addition, new, improved methods were developed.

### Assessing Cardiovascular Parameter Estimation Methods

The performance of the CV parameter estimation methods was assessed using the mean percentage error (MPE) and σ between estimated and reference CV parameter values for the two datasets of virtual subjects. In addition, Bland–Altman plots (48) were created to show the bias and limits of agreement (±1.96 standard deviation from the bias) between estimated and reference CV parameter values. For the 0-D dataset, reference values were obtained from the prescribed values used for each virtual subject (Table A1) (49–52). For the 1-D dataset, reference values for LVET, *P*_out_, and aortic root PWV were obtained from the prescribed values. *R*_T_ was calculated from the aortic root BP and flow waves using (15)

(1) $$R_{\text{T}}=\frac{\text{MBP}-P_{\text{out}}}{\bar{Q_{\text{in}}}},$$

where MBP is the mean blood pressure and $\bar{Q_{\text{in}}}$ is the mean blood flow. *C*_T_ and *Z*_0_ were extracted from aortic root BP and flow waves using the optimized three-element Windkessel model described in appendix A.2.

Two common clinical scenarios were considered when assessing CV parameter estimation methods for each dataset: carotid +, where the carotid BP wave was available, and carotid–, where only brachial DBP and SBP values were available (Fig. 1A). The 1-D dataset of virtual subjects was used to determine, for each scenario and CV parameter, the optimal (i.e., smallest MPE and σ) CV parameter estimation methods to be used by the cBP algorithms described in *Central Blood Pressure Estimation Algorithms.*

### Central Blood Pressure Estimation Algorithms

The three algorithms used to estimate cBP each consisted of two stages. First, CV parameters were estimated using the optimal CV parameter estimation methods. Second, a cBP wave was simulated using a computational model of arterial blood flow. We considered the following models: the two-element (15) and three-element (16) Windkessel models and a 1-D model of the thoracic aorta (11), referred to as 1D-Ao hereafter.

#### Two-element Windkessel (0-D) model

This model, referred to as 2-Wk hereafter, idealizes the arterial system as a reservoir of compliance *C*_T_. Blood flows into the reservoir from the heart, *Q*_in_(*t*), at a pressure *P*(*t*), encounters a resistance to flow, *R*_T_, and flows out into the vascular beds at a pressure *P*_out_ (Fig. 1C, *top*). The governing equation is

(2) $$\frac{\text{d}P}{\text{d}t}+\frac{P-P_{\text{out}}}{R_{\text{T}}C_{\text{T}}}=\frac{Q_{\text{in}}}{C_{\text{T}}},$$

which can be solved for *P*(*t*) using the integrating factor method,

(3) $$\begin{array}{l} P\left(t\right)=P_{\text{out}}+\left(P_{0}-P_{\text{out}}\right)e^{-\frac{t-t_{0}}{R_{\text{T}}C_{\text{T}}}}+\frac{e^{-\frac{t}{R_{\text{T}}C_{\text{T}}}}}{C_{\text{T}}}\int_{t_{0}}^{t}Q_{\text{in}}\left(t^{\prime }\right)e^{\frac{t^{\prime }}{e^{R_{\text{T}}C_{\text{T}}}}}\text{d}t^{\prime },\\ \;t\geq t_{0}, \end{array}$$

where *t*_0_ is the initial time and *P*_0_ = *P*(*t*_0_).

#### Three-element Windkessel (0-D) model

This model, referred to as 3-Wk hereafter, results from adding an impedance, *Z*_0_, in series to the 2-Wk model where *R*_T_ = *Z*_0_ + *R* (Fig. 1C, *middle*). *Z*_0_ is commonly known as the characteristic impedance and was initially introduced to represent the impedance of the aorta (26). The governing equation is

(4) $$\frac{\text{d}P}{\text{d}t}+\frac{P-P_{\text{out}}}{RC_{\text{T}}}=Z_{0}\frac{\text{d}Q_{\text{in}}}{\text{d}t}+\frac{\left(Z_{0}+R\right)Q_{\text{in}}}{RC_{\text{T}}},$$

which can be solved analytically for *P*(*t*) using the integrating factor method,

(5) $$\begin{array}{ll} P\left(t\right) & =P_{\text{out}}+\left(P_{0}-P_{\text{out}}-Z_{0}Q_{0}\right)e^{-\frac{t-t_{0}}{RC_{\text{T}}}}+Z_{0}Q_{\text{in}}\left(t\right)\\ +\frac{e^{-\frac{t}{RC_{\text{T}}}}}{C_{\text{T}}}\int_{t_{0}}^{t}Q_{\text{in}}\;\left(t^{\prime }\right)e^{\frac{t^{\prime }}{e^{RC_{\text{T}}}}}\text{d}t^{\prime },\;t\geq t_{0}, \end{array}$$

where *Q*_0_ = *Q*_in_(*t*_0_).

#### 1-D aortic model

This model uses the 1-D equations of blood flow in the network of compliant vessels shown in Fig. 1C (*bottom*) to compute cBP (11). The inputs to the model are *1*) the geometry (i.e., lengths and cross-sectional areas) of the thoracic aorta, including the supra-aortic arteries; *2*) flow waves at the ascending and descending aortas and, when available, each supra-aortic artery; and *3*) a peripheral BP measurement.

The 1-D and aortic coarctation datasets contained the vascular geometry and PWV data required to run the 1D-Ao algorithm. For the aortic coarctation dataset, the geometry of the thoracic aorta was extracted from MRI data using an in-house segmentation software (53, 54). Besides, since peripheral BP measurements were not available, the BP acquired invasively in the descending aorta was used instead. For the 1-D dataset, the geometry was extracted from the corresponding arterial segments. For both datasets, volumetric blood flow waves were obtained at the ascending (*Q*_in_, acquired as close to the aortic root as possible) and descending thoracic (*Q*_out_) aortas. *Q*_in_ and *Q*_out_ were used to calculate the pulse wave velocity, PWV, as described in Table 2.

*Q*_in_ was imposed as an inflow boundary condition at the aortic root and 3-Wk models were coupled to the outlet of each terminal 1-D model segment. The parameters of each outflow model $j,\;Z_{\text{0,W5k}}^{j},\;C_{\text{T,Wk}}^{j}$ and $R_{\text{Wk}}^{j}$ , were calculated using *Q*_in_, *Q*_out_, and the outflow distribution (OD) in the supra-aortic arteries, ${\text{OD}}_{\text{flow}}^{j}={\bar{Q}}_{\text{out}}^{j}/{\bar{Q}}_{\text{in}}$ , under the assumption that DBP, MBP, and *P*_out_ remain constant within large arteries (1). We used the following equations (11):

(6) $$Z_{0,\text{Wk}}^{j}=\frac{\rho \text{PWV}}{A_{\text{out}}^{j}},$$

(7) $$R_{\text{Wk}}^{j}=\frac{R_{\text{T}}}{{\text{OD}}^{j}}-Z_{0,\text{Wk}}^{j},$$

(8) $$C_{\text{T,Wk}}^{j}=\left(C_{\text{T}}-C_{\text{T,art}}\right)\frac{R_{\text{T}}}{R_{\text{Wk}}^{j}},$$

where *C*_T,art_ is the total compliance of the 1-D model arterial segments calculated as the sum of each segment compliance,

(9) $$C_{\text{T,art}}^{k}=\frac{{\bar{A}}^{k}L^{k}}{\rho {\text{PWV}}^{2}},$$

with ${\bar{A}}^{k}$ the average area and *L^k^* the length of the arterial segment *k*. When ${\bar{Q}}_{\text{out}}^{j}$ were unavailable at each outflow *j*, the difference between the mean values of *Q*_in_ and *Q*_out_ was distributed among the supra-aortic arteries proportionally to their outlet areas, $A_{\text{out}}^{j}$ , as ${\text{OD}}_{\text{area}}^{j}=\left({\bar{Q}}_{\text{in}}-{\bar{Q}}_{\text{out}}\right)A_{\text{out}}^{j}/\Sigma^{j}A_{\text{out}}^{j}$ .

### Assessing Central Blood Pressure Estimation Algorithms

The performance of each cBP estimation algorithm was assessed by comparing estimated cBP values with corresponding reference values in all clinical datasets and in the 1-D dataset. Performance was quantified using the μ and the σ of the errors for central diastolic (cDBP) and systolic (cSBP) blood pressure. In addition, the root mean square error (RMSE) between estimated and reference cBP waves was computed. Similar to *Assessing Cardiovascular Parameter Estimation Methods,* Bland–Altman plots were used to show the bias and limits of agreement between estimated and reference BP values. Finally, the correlation between estimated and reference cBP values was assessed using the coefficient of determination (*R* ^2^).

## ■ Results

### Assessment of CV Parameter Estimation Methods

The last two columns of Table 2 show mean percentage error (MPE) and standard deviation (σ) for all CV parameter estimation methods assessed in the two datasets of virtual subjects. MPE for the 1-D dataset was reduced by at least 40% if the carotid BP wave (carotid +) was used instead of brachial DBP and SBP values (carotid–).

Table 3 displays the methods that led to the smallest MPE for each clinical scenario and dataset. By using these optimal methods, all six CV parameters were calculated in less than 1 s for each virtual subject and in less than 1h for the entire 0-D or 1-D dataset using a Dell Precision M4800 laptop (Round Rock, TX).

**Figure 4:**
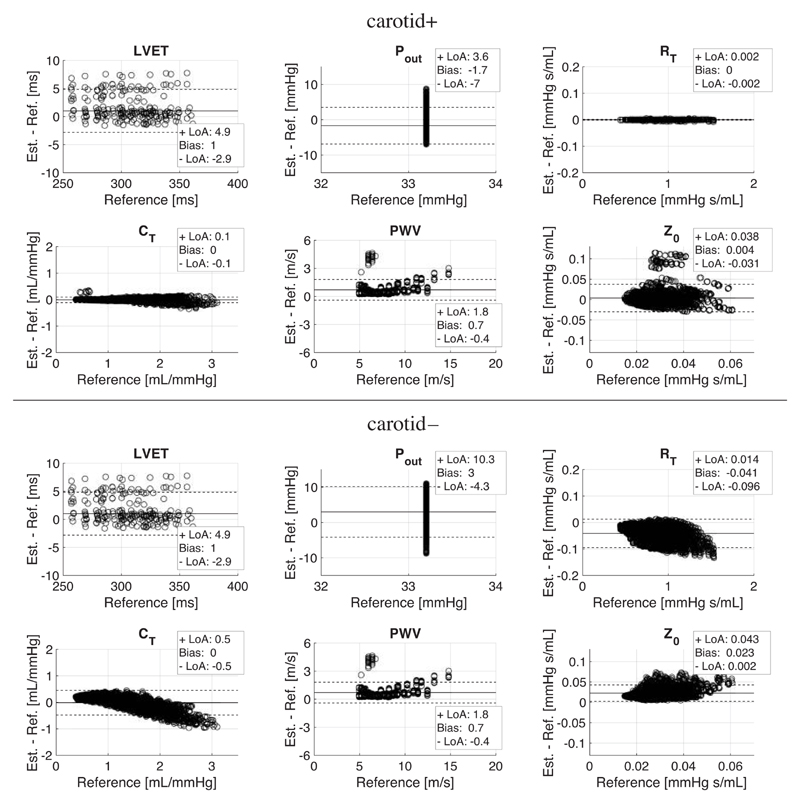
Bland–Altman plots for the optimal cardiovascular (CV) parameter estimation methods. They were obtained from all one-dimensional (1-D) dataset waves using the clinical scenarios carotid + (top) and carotid– *(bottom)*.

All parameters from the 0-D dataset were estimated with MPE < 2% in both clinical scenarios (Table 3, *top*). Figure 4 shows Bland–Altman plots for all CV parameters estimated using the optimal methods obtained from the 1-D dataset (Table 3, *bottom*). These methods were then used in the cBP estimation algorithms (see *Assessment of cBP Algorithms*).

For both scenarios, LVET, *P*_out_, *R*_T_, *C*_T_, and PWV were estimated without any considerable bias of their corresponding reference mean values (<6% for carotid + and <10% for carotid–). However, *Z*_0_ was overestimated with a much greater bias of its corresponding reference mean value (13% for carotid + and 82% for carotid–). The bias as a function of each CV parameter reference value remained approximately unchanged, with the exceptions of *P*_out_ (which had a singular reference value) and *C*_T_ for carotid– (whose absolute bias increased with increasing reference values). The same optimal methods were identified for PWV in both scenarios.

### Assessment of cBP Algorithms

The cBP algorithms used the optimal CV parameter estimation methods obtained from the 1-D dataset (Table 3, *bottom*). Table 4 shows the estimation errors for all three cBP algorithms, with each algorithm evaluated in four datasets for both clinical scenarios. In the 1-D dataset, RMSEs for carotid + (μ ± σ: < 3.4 ± 1.7 mmHg) were lower than those for carotid– (<5.1 ± 2.5 mmHg). In the clinical datasets, RMSEs were similar for both scenarios and larger than those obtained in the 1-D dataset. The 1D-Ao algorithm led to the smallest RMSEs in the 1-D (2.0 ± 1.0 mmHg) and aortic coarctation (6.4 ± 2.8 mmHg) datasets. The 3-Wk algorithm led to the smallest RMSEs in the normotensive (5.9 ± 2.4 mmHg) and hypertensive (5.7 ± 2.4 mmHg) datasets (these did not contain the aortic geometry data needed to run the 1D-Ao algorithm).

Overall, estimation errors for cDBP and cSBP were smaller in the 1-D dataset compared with the clinical datasets for all cBP algorithms and clinical scenarios. Furthermore, cDBP errors were smaller than cSBP errors for all algorithms, datasets, and scenarios. However, within each dataset and scenario, cDBP and cSBP errors changed considerably depending on the cBP algorithm used. For both clinical scenarios in the aortic coarctation and 1-D datasets, the 1D-Ao algorithm led to cSBP errors that were smaller or similar compared with the 0-D models (<2.2 ± 5.3mmHg vs. < 4.5 ± 5.9mmHg for the 1-D dataset; <2.1 ± 9.7 mmHg vs. <17.3 ± 7.9 mmHg for the aortic coarctation dataset). The 0-D algorithms performed similarly in both datasets and led to smaller cDBP errors than the 1D-Ao algorithm in the aortic coarctation dataset. *R* ^2^ correlation values between reference and estimated cBP calculated using the best-performing (i.e., 1-D aortic) algorithm and scenario in the 1-D dataset were: 0.834 for cDBP and 0.976 for cSBP (all *P* < 0.001). In the aortic coarctation dataset they were: 0.776 for cDBP and 0.903 for cSBP (all *P* < 0.001).

The normotensive and hypertensive datasets contained noninvasive reference cBP waves calculated by the SphygmoCor device using a transfer function. For carotid–, both 0-D models estimated cDBP and cSBP values with errors <6.0 ± 4.7 mmHg, though the 3-Wk algorithm led to smaller RMSEs in both datasets and scenarios. All errors for the 3-Wk algorithm were larger for carotid +. *R* ^2^ correlation values for these clinical datasets using the best-performing 0-D algorithm (i.e., 3-Wk) and scenarios were 0.949 for cDBP and 0.997 for cSBP (all *P* < 0.001).

An extended version of Table 4, which also contains errors for cMBP and cPP, is provided as Supplemental Table S1 (all Supplementary material is available at https://doi.org/10.5281/zenodo.3968540). Bland–Altman plots of cDBP, cSBP, cMBP, and cPP are also available (see Supplemental Figs. S1–S8). Supplemental Fig. S4 shows increases in the absolute bias for cSBP with increasing reference BP values in the 1-D, normotensive, and hypertensive datasets for carotid–. Remaining estimates were less affected by varying reference BP values.

Supplemental Figs. S9–S16 show individual cBP wave estimations by each cBP algorithm for a set of randomly chosen subjects in the 1-D dataset and for all subjects in the aortic coarctation, normotensive, and hypertensive datasets, in both clinical scenarios. Using a Dell Precision M4800 laptop, the 0-D algorithms took less than 1 s per patient to compute the cBP wave, whereas the 1D-Ao algorithm took less than 1 min (both times include the time required to calculate all patient-specific CV parameters).

## ■ Discussion

We have developed fast algorithms to estimate several clinically relevant hemodynamic parameters of the systemic circulation and reconstructed the cBP wave from noninvasive data. Our algorithms are based on physical phenomena occurring in the thoracic aorta and are patient specific for all physical parameters except for blood density and viscosity. We have tested them in several in silico and clinical datasets with a wide range of cBP wave morphologies. The 1D-Ao algorithm outperformed the 0-D algorithms at estimating cBP wave morphology when the aortic vascular geometry was available. Both 0-D models estimated cBP values with similar errors when only the aortic flow and peripheral BP waves were available, though the 3-Wk algorithm produced the smallest RMSEs. The aortic characteristic impedance was the most challenging CV parameter that needed to be estimated, limiting the ability of the 3-Wk algorithm to achieve smaller cBP errors. The novel Windkessel model dataset and optimized cBP algorithms are a valuable resource for developing and testing new, improved algorithms to estimate CV parameters and cBP waves.

### Cardiovascular Parameter Estimation Methods

Obtaining reliable in vivo reference values for the CV parameters required to estimate cBP is challenging. We, therefore, assessed the accuracy of several CV parameter estimation methods using datasets of virtual subjects for which theoretical reference values were either known exactly (all parameters for the 0-D dataset; LVET, *P*_out_, and PWV for the 1-D dataset) or could be estimated from the aortic BP and flow waves without measurement error (*R*_T_, *C*_T_, and *Z*_0_ for the 1-D dataset). Unlike the 0-D models, the 1-D model accounts for wave propagation phenomena and can capture high-frequency features of the pressure wave such as the first systolic shoulder, thus providing information that can be derived through pulse wave analysis. The 1-D dataset, therefore, provided the optimal combination of methods for the cBP algorithms and identified accurate methods for estimating CV parameters that, by themselves, can be used to assess cardiovascular function from noninvasive data available in the clinic.

Left ventricular ejection time (LVET) is a valuable metric of left ventricular performance both in health and disease (55). According to our results, it can be estimated accurately from the aortic flow wave using the novel LV 4 method (MPE ± σ: 0.3 ± 0.6%).

The physiological meaning and range of values of the asymptotic BP (*P*_out_) are still not fully understood (56). According to some studies, *P*_out_ is related to capillary and venous BP (57), though others argue this pressure is larger than the venous BP due to waterfall effects (58–60). We have found that estimation methods based on an exponential fit to the diastolic part of the BP wave outperformed those using a percentage of DBP (–5.1 ± 8.0% vs. 9.1 ± 11.0%).

Arterial resistance (*R*_T_) is also an important parameter for assessing small blood vessel function (61, 62). According to our results, calculation of *R*_T_ from peripheral DBP and SBP values underestimated reference *R*_T_ values by 5% on average. More accurate estimates could be obtained when using the whole peripheral BP wave (0.0 ± 0.1%).

Changes in arterial compliance (*C*_T_) can have important effects on the pulse wave, left ventricular dynamics, cardiac output, and the ratio of systolic to diastolic flow into capillary beds (63). Our proposed optimized 3-Wk method for estimating *C*_T_ led to a MPE = –0.8 ± 4.2%, outperforming existing methods. Similar to the study by Stergiopulos et al. (64), we found MPE < 12% for the diastolic decay, area, and two-area methods, though our MPE for the pulse pressure method was higher (27% vs. 17%).

Pulse wave velocity (PWV) provides a direct measure of aortic stiffness and is an independent predictor of cardiovascular risk (65, 66). We found that methods for estimating PWV that used the ascending and descending aorta flows outperformed those using the carotid and femoral BP waves, in agreement with the study by Obeid et al. (67), which also involved in silico data and theoretical reference PWV values.

Aortic characteristic impedance (*Z*_0_) is directly related to aortic stiffness (40, 68). In the 1-D dataset, the PQ-loop methods led to smaller MPE (13.4%) than other methods (>37.1%), including those with MPE < 3% when run on the 0-D dataset. Most methods involving BP and flow waves require these to be measured simultaneously at the same location, but in this study, BP was taken from the periphery and combined with the aortic flow wave, resulting in large MPE for the 1-D dataset (>13.4%). PQ-loop methods only require a linear proportionality between aortic BP and flow in early systole, which, according to our results, is maintained between peripheral BP and aortic flow. In fact, BP and flow morphology in early systole are mainly dictated by the propagation of a pulse wave traveling from the heart to the periphery, with the backward-traveling wave having little influence (69). This observation led to the derivation of the novel method Z4, which provided the smallest MPE for carotid– (82.3 ± 32.6%).

Finally, we note that all CV parameters were estimated individually from the clinical data. However, due to the interdependence between some CV parameters (e.g., *R*_T_ and *P*_out_), performance may be improved via simultaneous or iterative estimation, as suggested by Parragh et al. (56), though this was beyond the scope of our study.

### Central Blood Pressure Algorithms

We have developed algorithms that estimate the cBP wave from noninvasive, patient-specific measurements by using 0-D and 1-D blood flow modeling. 0-D models were chosen for their simplicity and low number of CV parameters that have to be estimated. The 1D-Ao model was chosen because it captures pulse wave propagation phenomena, though at the expense of a much larger number of parameter estimations. Only the thoracic aorta was simulated using 1-D model segments since cardiac MRI usually provides vessel anatomy and blood flow in the upper part of the aorta only. Furthermore, previous work has shown that it is possible to reduce the topological complexity of the arterial network and, hence, the number of parameters to be estimated, while sufficiently capturing relevant BP values such as cSBP and cPP (70, 71).

We tested the cBP algorithms in several clinical datasets to cover a wide range of cBP wave morphologies, including those seen in hypertensive subjects and in normotensive subjects under the effect of four inotropic and vasoactive drugs that significantly affect BP wave morphology (72). When the aortic vascular geometry was available, the 1D-Ao algorithm outperformed the 0-D algorithms at estimating cBP wave morphology as well as cSBP values, leading to RMSEs < 2.0 ± 1.0 mmHg in the 1-D dataset and <6.4 ± 2.8 mmHg in the aortic coarctation dataset. When the aortic vascular geometry was unavailable, the three-element 0-D algorithm achieved RMSEs < 2.0 ± 1.7 mmHg for in silico data and <5.9 ± 2.4 mmHg for clinical data from the normotensive and hypertensive datasets.

Relative errors for cBP estimates were smaller in the 1-D dataset than in the clinical datasets since all hemodynamic data in the former were free of measurement error and inconsistencies that are inherent to clinical datasets (e.g., heart rate differences between pressure and flow waves) (11). Therefore, results obtained from the 1-D dataset provided a theoretical lower bound of cBP errors to be expected when analyzing clinical datasets.

Recent (2017) clinical guidelines for the validation of noninvasive cBP devices propose a mean absolute difference of ≤5mmHg with σ ≤ 8 mmHg compared with the reference cSBP (19). The potential of the algorithms used in this study to achieve mean absolute differences that are almost within recommended values in clinical cohorts with either invasive reference cBP values (aortic coarctation dataset) or cBP values calculated by the widely used SphygmoCor device (normotensive and hypertensive datasets) has been shown. On the one hand, the 1D-Ao algorithm achieved mean absolute differences <2.1 ± 9.7 mmHg for cSBP values in the aortic coarctation dataset for both scenarios. On the other hand, the 0-D models achieved mean absolute differences <8.6 ± 5.0 mmHg in the normotensive dataset and <8.0 ± 10.6 mmHg in the hypertensive dataset. Furthermore, the lower-bound RMSEs obtained when testing all algorithms in the measurement error-free 1-D dataset were even smaller (<3.4 ± 1.7 mmHg for carotid + and <5.1 ± 2.5 mmHg for carotid–), suggesting that our algorithms’ performance could be within recommended values if measurement error and data inconsistencies could be reduced further during data acquisition.

Central BP estimates for some subjects in the normotensive and hypertensive datasets showed large errors (>50 mmHg). These subjects had noisy ultrasound velocity time integral (VTI) waves (used to calculate aortic flow waves) characterized by either an extended diastolic phase (resulting in LVET > 50% of the cardiac cycle duration) or a large second peak after the systolic peak. Both artifacts could explain the smaller cBP estimation errors for the 0-D models in the more challenging carotid– scenario compared with carotid +.

A review of methods to estimate cSBP from arterial pulse waves (73) found a mean error (95% confidence interval) of –1.1 (–2.8 to 0.7) mmHg when calibrated using invasive BP values and a mean error of –5.8 (–7.8 to 3.8) mmHg when calibrated using noninvasive BP values. In our study, the 1D-Ao algorithm was found to have mean errors of 0.0 (–6.0 to 6.0) mmHg when calibrated using an invasive BP waveform (carotid + scenario in the aortic coarctation dataset) –2.1 (–7.8 to 3.6) mmHg when using invasive BP values (carotid–scenario in the aortic coarctation dataset), and the 2-Wk algorithm was found to have mean errors when calibrated noninvasively of –3.3 (–3.9 to –2.7) mmHg (carotid– scenario in the normotensive dataset) and –5.5 (–6.1 to –4.9) mmHg (carotid– scenario in the hypertensive dataset). Thus, the mean cSBP error provided by the models presented in this study was comparable with those observed in previous studies of cSBP estimation methods. Unlike transfer function methods, our proposed cBP algorithms do not need to be trained on existing clinical datasets and make no assumptions regarding generalizability, since they simulate patient-specific hemodynamic phenomena occurring in the aorta where cBP is calculated. This may be advantageous when applying these algorithms to the wider population, including patients suffering from a range of CV diseases or under pharmacological treatment. However, a direct comparison against such techniques was not possible due to the lack of required data and corresponding devices.

### Limitations

The peripheral pressure wave (*P*) required by the cBP algorithms was measured invasively in the descending aorta in the aortic coarctation dataset. Since this may give the algorithms an advantage compared with noninvasive methods using cuff or tonometry measurements, the 1-D dataset—which contained *P* at the required peripheral locations—was also used for the final cBP algorithm assessment. In the normotensive and hypertensive datasets, since invasive reference cBP measurements were not available, noninvasive measurements were obtained using the SphygmoCor device. Although these measurements are not exactly equivalent to invasive cBP, they allowed us to compare the performance of the cBP algorithms to a widely used noninvasive device. We note that the aortic coarctation dataset contained data from subjects—in the future, further studies should verify the conclusions presented here using additional data with invasive reference measurements.

### Perspectives

Patients with cardiovascular disease would benefit from an accurate noninvasive assessment of their cBP. Our approach removes the risk of complications due to cardiac catheterization and allows for a more regular assessment of a patient’s cBP, due to its noninvasive nature. Moreover, it is relatively quick: it only takes a few seconds (when using the 0-D algorithms) or a few minutes (when using 1D-Ao algorithm) to compute cBP on a Dell Precision M4800 laptop. The 1-D algorithm is particularly relevant in clinical cardiology, where cardiac MRI is increasingly used. Indeed, the detailed geometric and flow data obtained using MRI can lead to important improvements in noninvasive cBP estimation, which could lead to a better adaption in clinical practice. In addition, the 0-D algorithms can be used in combination with US scans to obtain patient-specific cBP estimates.

The novel Windkessel model dataset and optimized cBP algorithms are freely available (see DATA ACCESS STATEMENT) to develop and test new, improved algorithms for estimating CV parameters and cBP waves.

## Conclusion

We have presented freely available, fast, patient-specific algorithms to estimate clinically relevant CV parameters and reconstruct the cBP wave from the aortic flow wave, using non-invasive data and patient-specific models of aortic blood flow. We have tested our algorithms against a wide range of cBP morphologies from several clinical datasets, one of which included catheter cBP waves. Finally, we have shown the potential of our algorithms to estimate cBP values within guideline-recommended values. Our approach could improve CV function assessment in clinical cohorts for which aortic ultrasound or magnetic resonance imaging data are available.

## Supplementary Material

Supplementary material

Appendix

## Figures and Tables

**Table 1:** Datasets’ characteristics

	Dataset				
	Ao Co	Normotensive	Hypertensive	0-D Dataset	1-D Dataset
Subjects (males)	10 (9)	13 (10)	158 (80)	15,582 (N/A)	4,064 (N/A)
Age, yr	20.8 ± 9.1	48.4 ± 9.4	46.2 ± 16.7	N/A	50 ± 17.1^†^
DBP, mmHg	53.2 ± 8.9	68.4 ± 10.4^*a*^	81.8 ± 12.8^*a*^	64.6 ± 9.0	75.3 ± 7.3
MBP, mmHg	69.3 ± 9.7	85.6 ± 12.1^*b*^	102.0 ± 15.8^*b*^	83.9 ± 11.2	94.2 ± 6.7
pSBP, mmHg	82.0 ± 15.2	111.4 ± 17.3^*c*^	129.6 ± 22.6^*c*^	117.6 ± 21.3	119.3 ± 11.4
cSBP, mmHg	93.7 ± 11.9	107.2 ± 17.3	126.4 ± 22.2		110.4 ± 12.5
pPP, mmHg	30.6 ± 13.0	43.2 ± 12.2	48.2 ± 16.0	52.9 ± 16.9	46.5 ± 14.1
cPP, mmHg	40.5 ± 12.7	38.8 ± 11.0	44.6 ± 15.4		35.1 ± 15.3
SV, mL	57.4 ± 29.9	100.6 ± 35.3	83.3 ± 32.8	88.4 ± 12.2	60.3 ± 12.3
HR, beats/min	65.1 ± 14.4	62.2 ± 11.2	65.5 ± 10.4	68.8 ± 11.3	75.9 ± 9.3
CO, L/min	3.6 ± 1.7	6.2 ± 2.5	5.3 ± 1.9	6.1 ± 1.3	4.6 ± 1.1

Ao Co, aortic coarctation dataset; CO, cardiac output; cPP, central pulse pressure; cSBP, central systolic blood pressure; DBP, diastolic blood pressure; HR, heart rate; MBP, mean blood pressure; pPP, peripheral pulse pressure; pSBP, peripheral systolic blood pressure; SV, stroke volume.

† Age ranges from 25 to 75 yr, with 10-yr intervals.

a Brachial oscillometric measurement.

b Radial tonometry measurement.

c Carotid tonometry measurement.

**Table 2:** CV parameter estimation methods assessed in this study

Parameter	Description	Sce	Ref	Abb	Percentage Error, %	
					0-D dataset	1-D dataset
Left ventricular ejection time, LVET	d*P*/dt analysis, 1	+	(32)	LV1	‡	0.4 ± 1.0
	d*P*/d*t* analysis, 2	+	(37)	LV2	–12.4 ± 0.1	–5.7 ± 4.1
	0.37 $\sqrt{T}$	+, –	(31)	LV3	26.1 ± 8.5	6.9 ± 8.1
	*Q* analysis	+, –	†	LV4	0.1 ± 0.2	0.3 ± 0.6
Outflow pressure, *P*_out_	Diastolic decay fit, 1	+	(15, 26)	OP1	0.0 ± 0.0	–5.1 ± 8.0
	Diastolic decay fit, 2	+	(15, 44)	OP2	0.0 ± 0.0	–10.5 ± 7.5
	0.5 DBP	+, –	†	OP3	1.6 ± 16.9	9.1 ± 11.0
	0.7 DBP	+, –	(56)	OP4	42.3 ± 23.6	52.7 ± 15.4
Arterial resistance, *R*_T_	$\left(\text{MBP }-\;P_{\text{out}}\right)/\bar{Q}$	+	(15)	AR1	0.0 ± 0.0	0.0 ± 0.1
	$\left(\text{DBP }+0.4\text{PP }-\;P_{\text{out}}\right)/\bar{Q}$	+, –	(22, 15)	AR2	0.7 ± 5.7	–4.9 ± 2.9
Arterial compliance, *C*_T_	2-point diastolic decay	+	(15)	AC1	–0.1 ± 0.0	–6.5 ± 4.9
	Diastolic decay fit, 1	+	(15)	AC2	0.0 ± 0.0	–6.6 ± 3.3
	Diastolic decay fit, 2	+	(15, 44)	AC3	0.0 ± 0.0	–10.2 ± 5.0
	Area method	+	(27, 41, 26)	AC4	–10.0 ± 4.1	–11.4 ± 4.6
	Two-area method	+	(43, 26)	AC5	–10.0 ± 4.1	–7.1 ± 7.1
	DBP method	+, –	†	AC6	–1.5 ± 4.1	–17.3 ± 7.5
	PP method	+, –	(25, 26)	AC7	–0.1 ± 0.2	–27.6 ± 11.6
	SV/PP	+, –	(27)	AC8	–13.8 ± 20.3	4.9 ± 18.4
	Optimized 3-Wk	+	†	AC9	0.0 ± 0.3	–0.8 ± 4.2
Pulse wave velocity, PWV	Foot-to-foot: *Q*_Ao_	+, –	(35)	PV1	–	8.2 ± 6.0
	Foot-to-foot: *P*_c-f_	+^a^	(35)	PV2	–	27.8 ± 10.8
	Least-squares: *Q*_Ao_	+, –	(35)	PV3	–	–12.7 ± 8.3
	Least-squares: *P*_c-f_	+^a^	(35)	PV4	–	43.0 ± 36.0
	Sum of squares	+	(34)	PV5	–	33.2 ± 17.2
Characteristic impedance, *Z*_0_	Frequency methods	+	(29, 33, 23, 36, 38, 40, 24, 42)	Z1	2.5 ± 2.1	64.6 ± 44.3
	PQ-loop methods	+	(23, 28, 45)	Z2	0.2 ± 1.4	13.4 ± 56.6
	0.05 *R*_T_	+, –	(39, 46)	Z3	–1.5 ± 40.8	133.8 ± 66.7
	(MBP - DBP)/*Q*_max_	+, –	†	Z4	–38.7 ± 12.4	82.3 ± 32.6
	ρPWV/A	+, –	(47)	Z5	–	90.4 ± 18.1
	Optimized 3-Wk	+	†	Z6	–0.1 ± 0.7	37.1 ± 20.0

Errors are presented as the means ± SD of the percentage error between estimated and reference CV parameter values. *A*, aortic root cross-sectional area; Abb, coded abbreviations used to refer to each method; DBP, diastolic blood pressure; MBP, mean blood pressure; *P*, peripheral BP waveform; PP, pulse BP values from *P*; *P*_c-f_, carotid–femoral blood BP wave pair; *Q*, aortic root flow waveform; $\bar{Q}$ , mean value of *Q* over *T*; *Q*_Ao_, ascending and descending aorta flow wave pair; *Q*_max_, peak aortic flow; Ref, references; Sce, clinical scenarios (+: carotid +, carotid–); SV, stroke volume; *T*, duration of cardiac cycle; 3-Wk, 3-element Windkessel; ρ, blood density. Performance was assessed in two clinical scenarios (carotid +: carotid BP wave available; carotid–: only brachial DBP and SBP available) using the 0-D and 1-D datasets (Fig. 1A).

† Newly proposed methods (described in appendix B).

‡ BP waves from the 0-D dataset do not present a second systolic peak as required by LV1.

a BP waves at the carotid and femoral arteries required.

**Table 3:** Optimal CV parameter estimation methods for both datasets and clinical scenarios

		Optimal CV Parameter Estimation Methods (MPE, %)					
Dataset	Sce	LVET	*P*_out_	*R*_T_	*C*_T_	PWV	*Z*_o_
0-D dataset	+	LV4 (0.3)	OP1/2 (0.0)	AR1 (0.0)	AC2/3 (0.0)	N/A	Z6 (–0.1)
	–		OP3 (1.6)	AR2 (0.7)	AC7 (–0.1)	N/A	Z3 (–1.5)
1-D dataset	+	LV4 (0.3)	OP1 (–5.1)	AR1 (0.0)	AC9 (–0.8)	PV1 (8.2)	Z2 (13.4)
	–		OP3 (9.1)	AR2 (–4.9)	AC8 (4.9)	PV1 (8.2)	Z4 (82.3)

*C*_T_, arterial compliance; LVET, left-ventricular ejection time; MPE, mean percentage error for the entire dataset; *P*_out_, outflow BP; PWV, pulse wave velocity; *R*_T_, arterial resistance; Sce, clinical scenarios (+: carotid +, –: carotid–); *Z*_0_, characteristic impedance. The abbreviations for each method (e.g., LV4) correspond to those described in Table 2.

**Table 4:** Performance of cBP estimation algorithms

			Estimation Error (μ ± σ), mmHg		
Dataset	Scenario	Algorithm	cDBP	cSBP	RMSE
1-D dataset	Carotid +	2-Wk	1.2 ± 0.7	1.0 ± 0.8	3.4 ± 1.1
		3-Wk	0.1 ± 1.0	1.8 ± 1.9	2.0 ± 1.7
		1 D-Ao	0.1 ± 1.1	2.2 ± 1.8	2.0 ± 1.0
	Carotid –	2-Wk	0.8 ± 1.5	–4.5 ± 5.9	5.0 ± 2.5
		3-Wk	–2.6 ± 0.8	–0.2 ± 4.7	5.1 ± 2.0
		1 D-Ao	–1.5 ± 1.2	–1.7 ± 5.3	4.2 ± 2.1
Aortic Coarctation	Carotid +	2-Wk	0.8 ± 3.1	–15.7 ± 7.2	10.1 ± 3.9
		3-Wk	0.2 ± 2.8	–15.4 ± 7.4	8.0 ± 3.2
		1 D-Ao	–3.4 ± 4.8	–0.0 ± 9.7	6.4 ± 2.8
	Carotid –	2-Wk	–1.5 ± 2.4	–17.3 ± 7.9	10.9 ± 4.3
		3-Wk	–1.8 ± 2.5	–17.2 ± 7.9	8.4 ± 3.6
		1 D-Ao	–6.1 ± 2.8	–2.1 ± 9.2	7.8 ± 3.3
Normotensive	Carotid +	2-Wk	4.7 ± 1.9	–8.6 ± 5.0	10.3 ± 3.0
		3-Wk	–4.4 ± 3.5	13.4 ± 13.4	8.6 ± 5.5
	Carotid –	2-Wk	–0.1 ± 0.5	–3.3 ± 3.5	11.0 ± 3.5
		3-Wk	0.2 ± 0.5	–3.7 ± 4.0	5.9 ± 2.4
Hypertensive	Carotid +	2-Wk	5.0 ± 3.2	–8.3 ± 6.3	10.6 ± 4.1
		3-Wk	–2.9 ± 3.6	8.0 ± 10.6	7.1 ± 4.2
	Carotid –	2-Wk	–0.3 ± 0.8	–5.5 ± 4.0	11.1 ± 4.2
		3-Wk	0.0 ± 0.6	–6.0 ± 4.7	5.7 ± 2.4

Results are presented as mean (μ) and standard deviation (σ) errors between estimated and reference values of cDBP and cSBP. The RMSE between estimated and reference cBP waves is shown in the last column. Each cBP algorithm was assessed in four datasets and two clinical scenarios: carotid + (peripheral BP wave available) and carotid– (only peripheral SBP and DBP available). cDBP, central diastolic blood pressure; cSBP, central systolic blood pressure; RMSE, root mean square error.
