# Supplementary material for: Estimating central blood pressure from aortic flow: development and assessment of algorithms
Source: Am J Physiol Heart Circ Physiol. Author manuscript; Available in PMC 2022 Mar 26. (PMC7612539; doi:10.1152/ajpheart.00241.2020)
Supplement: Supplementary material [file EMS143859-supplement-Supplementary_material.pdf]

DATA SUPPLEMENT
Estimating central blood pressure from aortic flow: development and assessment of algorithms

Jorge Mariscal-Harana[1], Peter H. Charlton[1], Samuel Vennin[1,2], Jorge Aramburu[3], Mateusz C. Florkow[1,4], Arna van Engelen[1], Torben Schneider[5], Hubrecht de Bliek[6], Bram Ruijsink[1,7], Israel Valverde[1,8], Philipp Beerbaum[9], Heynric Grotenhuis[10], Marietta Charakida[1], Phil Chowienczyk[2], Spencer Sherwin[11], and Jordi Alastruey[1,12]

[1] Department of Biomedical Engineering, School of Biomedical Engineering and Imaging Sciences, King's College London, King's Health Partners, SE1 7EH, UK
[2] Department of Clinical Pharmacology, King's College London, King's Health Partners, London, SE1 7EH, UK
[3] Universidad de Navarra, TECNUN Escuela de Ingenieros, 20018 Donostia-San Sebastián, Spain
[4] Philips Research, Cambridge, UK
[5] Philips Healthcare UK, Philips Centre, Guildford Business Park, Guildford, Surrey, GU2 8HX, UK
[6] HSDP Clinical Platforms, Philips Healthcare, Eindhoven, The Netherlands
[7] Department of Cardiology, University Medical Centre Utrecht, Utrecht, The Netherlands
[8] Cardiovascular Pathophysiology, Institute of Biomedicine of Seville, University Hospital of Virgen del Rocío, University of Seville, CIBERCV, CSIC, Seville, Spain.
[9] Department of Pediatric Cardiology and Intensive Care, Hannover Medical School, Hannover, Germany
[10] Department of Pediatric Cardiology, University Medical Center Utrecht / Wilhelmina Children's Hospital, Utrecht, The Netherlands.
[11] Department of Aeronautics, South Kensington Campus, Imperial College London, SW7 2AZ, UK
[12] Institute of Personalized Medicine, Sechenov University, Moscow, Russia

E-mail: `jorge.mariscal_harana@kcl.ac.uk`

1. Performance of cBP estimation algorithms

Table S1: Performance of cBP estimation algorithms. Results are presented as mean (μ) and standard deviation (σ) errors between estimated and reference values of $cDBP$, $cMBP$, $cSBP$, and cPP. The RMSE between estimated and reference cBP waves is shown in the last column. Each cBP algorithm was assessed in four datasets and two clinical scenarios: 'carotid+' (peripheral BP wave available) and 'carotid−' (only peripheral SBP and DBP available).

			**Estimation error ($\mu \pm \sigma$) [mmHg]**				
**Dataset**	**Sce**	**Algo**	$cDBP$	$cMBP$	$cSBP$	cPP	RMSE
**1-D dataset**	+	2-Wk	1.2 ± 0.7	0.1 ± 0.1	1.0 ± 0.8	-0.2 ± 1.3	3.4 ± 1.1
		3-Wk	0.1 ± 1.0	0.1 ± 0.1	1.8 ± 1.9	1.7 ± 2.8	2.0 ± 1.7
		1D-Ao	0.1 ± 1.1	0.2 ± 0.6	2.2 ± 1.8	2.1 ± 2.3	2.0 ± 1.0
	−	2-Wk	0.8 ± 1.5	-3.0 ± 1.9	-4.5 ± 5.9	-5.3 ± 7.2	5.0 ± 2.5
		3-Wk	-2.6 ± 0.8	-2.9 ± 1.9	-0.2 ± 4.7	2.4 ± 5.2	5.1 ± 2.0
		1D-Ao	-1.5 ± 1.2	-2.9 ± 1.9	-1.7 ± 5.3	-0.2 ± 6.3	4.2 ± 2.1
**Aortic Coarctation**	+	2-Wk	0.8 ± 3.1	-3.4 ± 2.8	-15.7 ± 7.2	-16.4 ± 8.5	10.1 ± 3.9
		3-Wk	0.2 ± 2.8	-3.5 ± 2.8	-15.4 ± 7.4	-15.6 ± 8.6	8.0 ± 3.2
		1D-Ao	-3.4 ± 4.8	-2.5 ± 2.6	-0.0 ± 9.7	3.4 ± 10.7	6.4 ± 2.8
	−	2-Wk	-1.5 ± 2.4	-5.5 ± 2.8	-17.3 ± 7.9	-15.8 ± 9.2	10.9 ± 4.3
		3-Wk	-1.8 ± 2.5	-5.7 ± 2.9	-17.2 ± 7.9	-15.4 ± 9.1	8.4 ± 3.6
		1D-Ao	-6.1 ± 2.8	-5.3 ± 3.0	-2.1 ± 9.2	4.1 ± 10.6	7.8 ± 3.3
**Normotensive**	+	2-Wk	4.7 ± 1.9	0.3 ± 0.1	-8.6 ± 5.0	-13.3 ± 6.2	10.3 ± 3.0
		3-Wk	-4.4 ± 3.5	-0.9 ± 0.7	13.4 ± 13.4	17.9 ± 16.5	8.6 ± 5.5
	−	2-Wk	-0.1 ± 0.5	0.2 ± 2.2	-3.3 ± 3.5	-3.2 ± 3.4	11.0 ± 3.5
		3-Wk	0.2 ± 0.5	-0.2 ± 2.1	-3.7 ± 4.0	-3.9 ± 4.1	5.9 ± 2.4
**Hypertensive**	+	2-Wk	5.0 ± 3.2	0.3 ± 0.1	-8.3 ± 6.3	-13.3 ± 9.0	10.6 ± 4.1
		3-Wk	-2.9 ± 3.6	-0.9 ± 1.0	8.0 ± 10.6	11.0 ± 13.4	7.1 ± 4.2
	−	2-Wk	-0.3 ± 0.8	-1.1 ± 2.0	-5.5 ± 4.0	-5.2 ± 4.1	11.1 ± 4.2
		3-Wk	0.0 ± 0.6	-1.5 ± 2.0	-6.0 ± 4.7	-6.0 ± 4.9	5.7 ± 2.4

Abbreviations: **Sce**: clinical scenarios (+: 'carotid+', −: 'carotid−'); **Algo**: cBP estimation algorithms (2-Wk: 2-element Windkessel, 3-Wk: 3-element Windkessel, and 1D-Ao: 1-D aortic).

2. Bland-Altman plots assessing cBP estimates

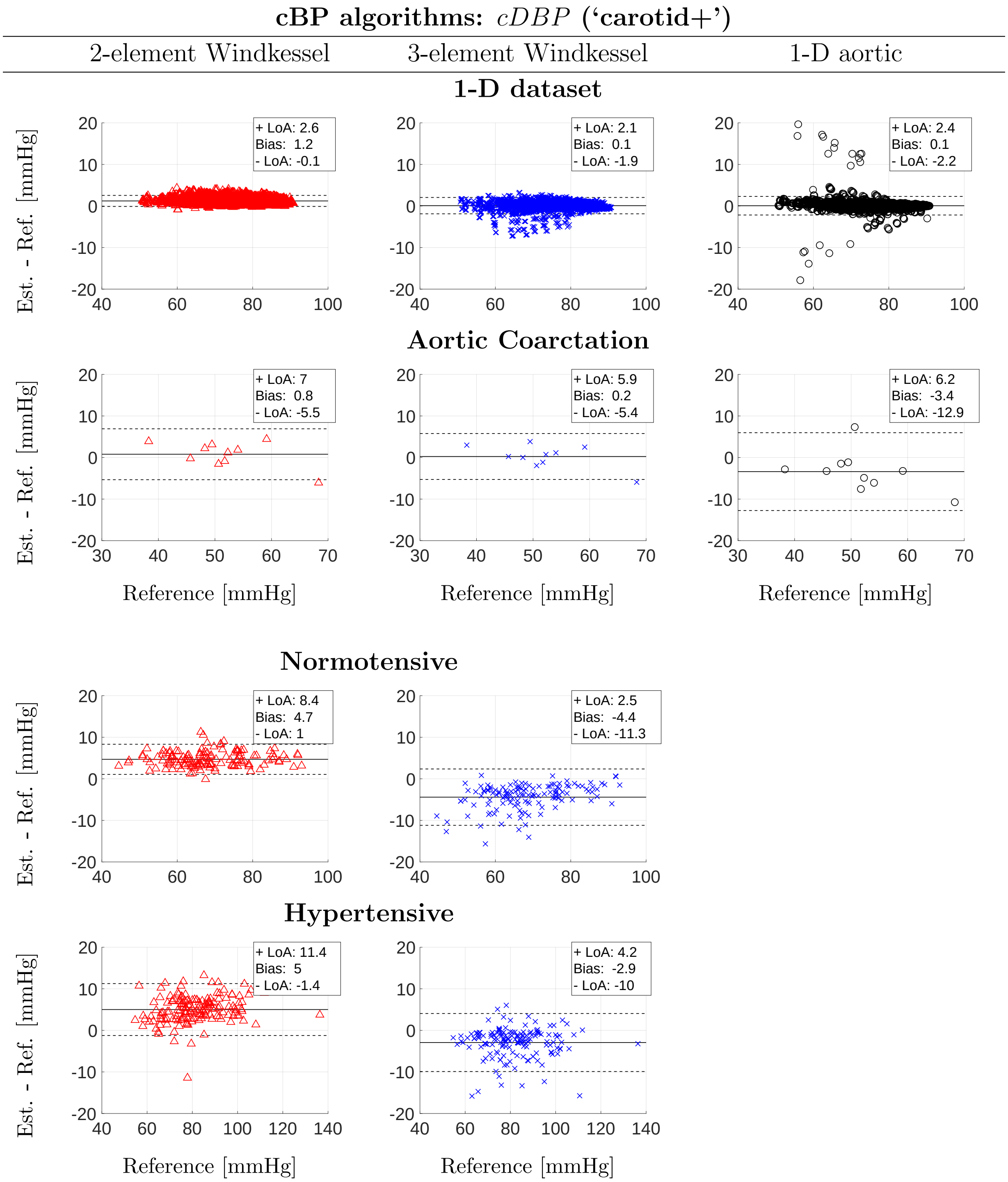

Figure S1: Bland-Altman plots for cDBP estimation for 'carotid+'. The 2-element Windkessel (△), 3-element Windkessel (×) and 1-D aortic (○) algorithms were assessed in the 1-D (top), 'Aortic Coarctation' (second row), 'Normotensive' (third row), and 'Hypertensive' (bottom) datasets. y-axes are estimated minus reference cDBP values.

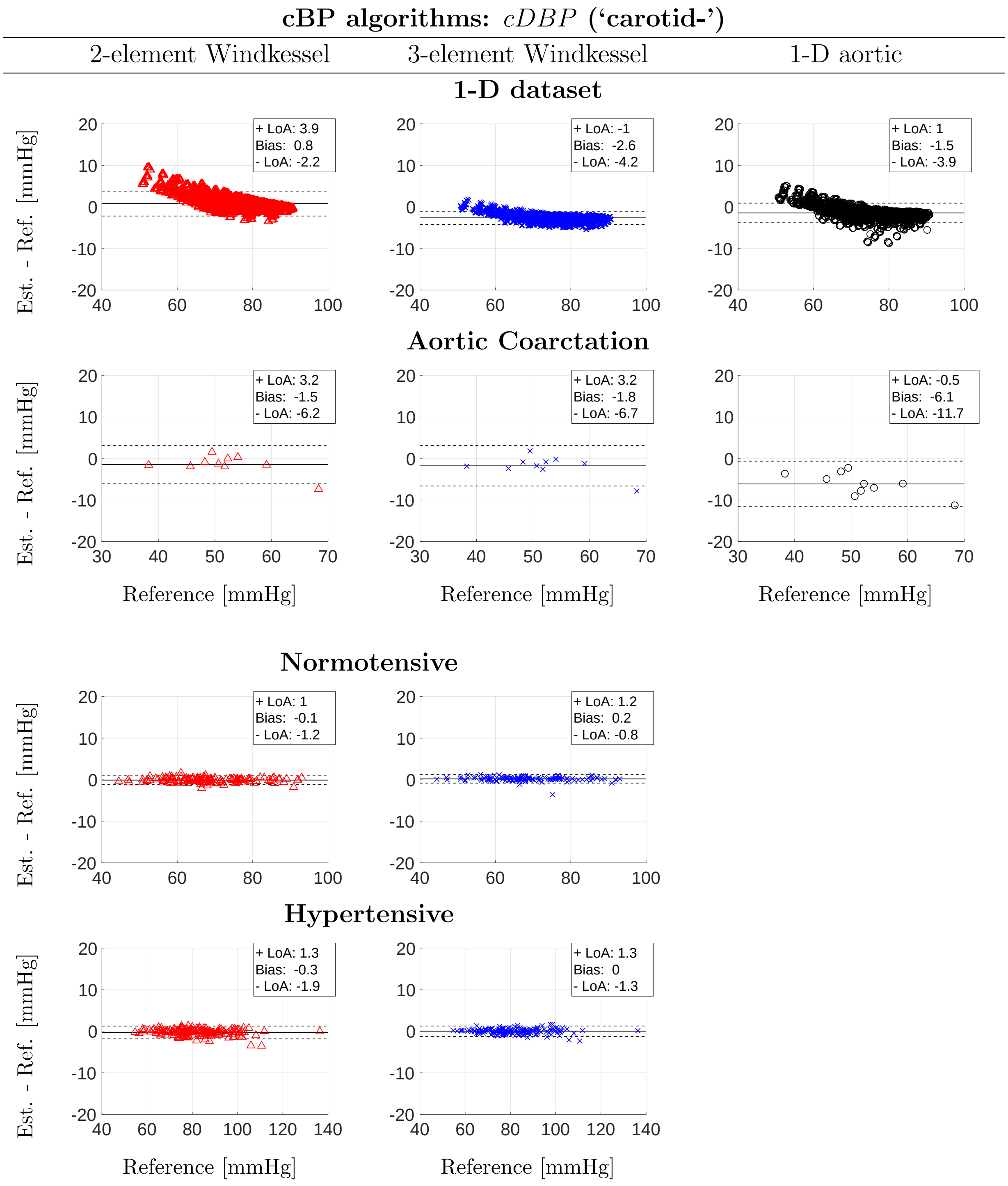

Figure S2: Bland-Altman plots for cDBP estimation for 'carotid-'. The 2-element Windkessel (△), 3-element Windkessel (×) and 1-D aortic (◯) algorithms were assessed in the 1-D (top), 'Aortic Coarctation' (second row), 'Normotensive' (third row), and 'Hypertensive' (bottom) datasets. y-axes are estimated minus reference cDBP values.

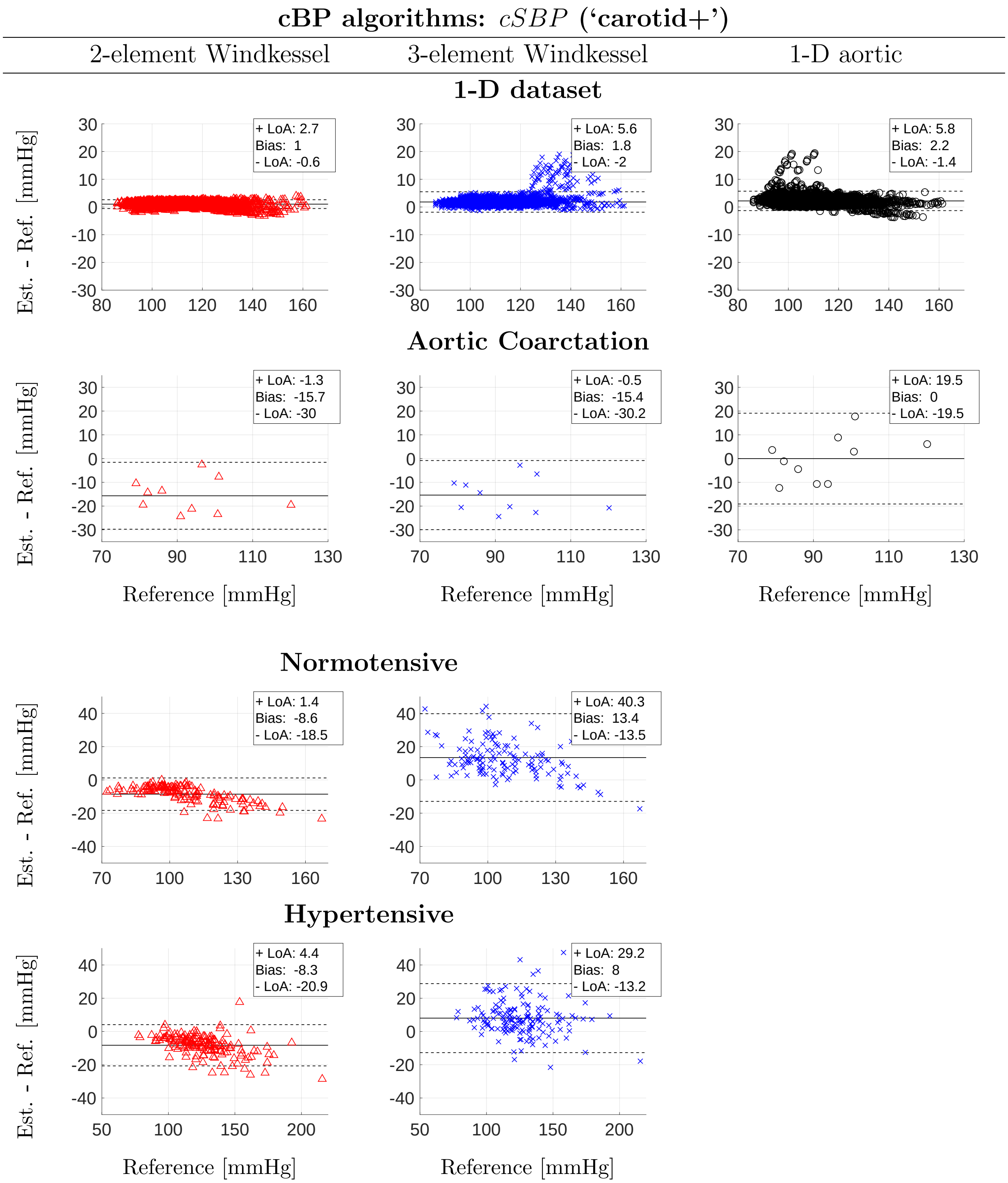

Figure S3: Bland-Altman plots for cSBP estimation for 'carotid+'. The 2-element Windkessel (△), 3-element Windkessel (×) and 1-D aortic (◯) algorithms were assessed in the 1-D (top), 'Aortic Coarctation' (second row), 'Normotensive' (third row), and 'Hypertensive' (bottom) datasets. y-axes are estimated minus reference cSBP values.

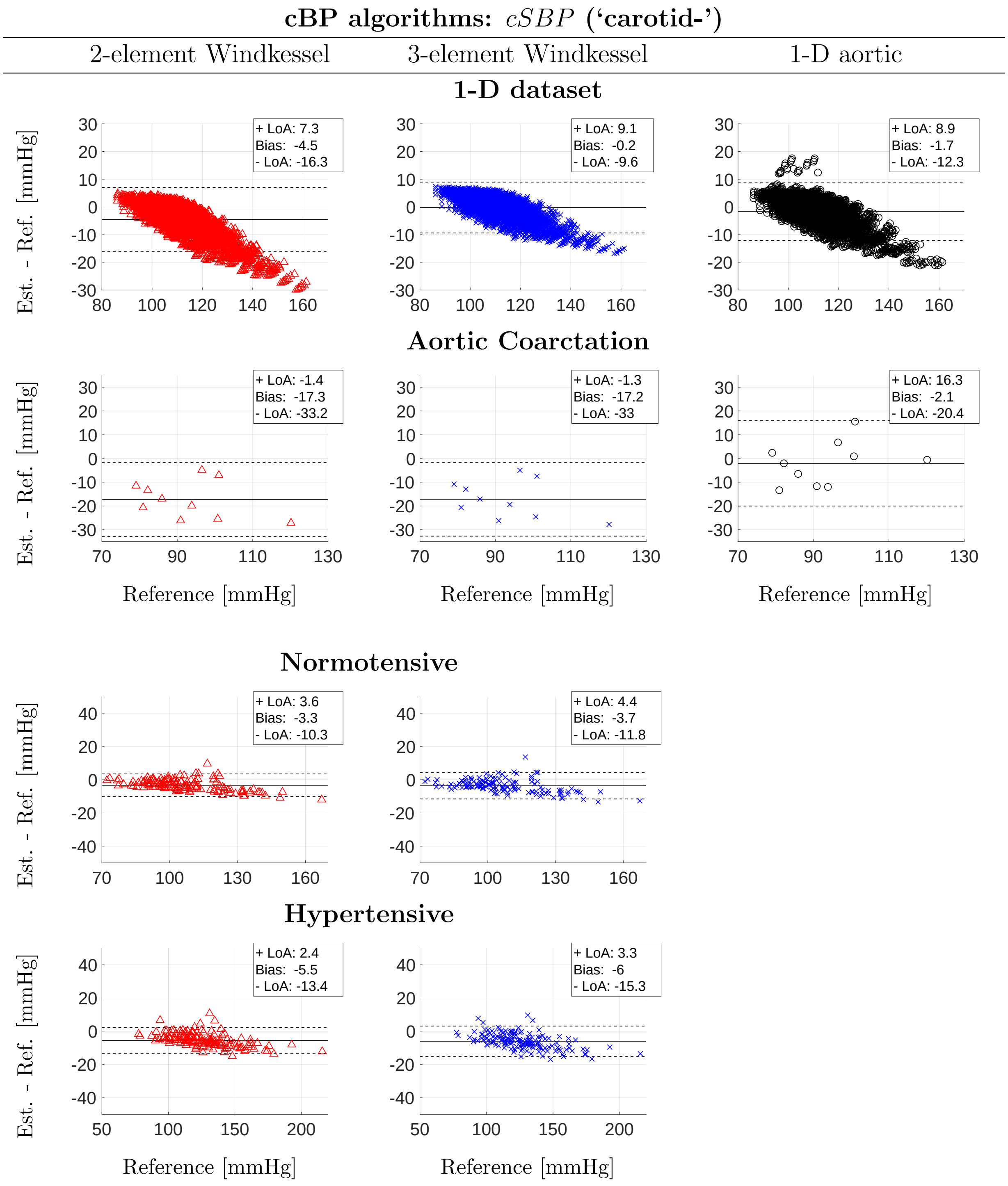

Figure S4: Bland-Altman plots for cSBP estimation for 'carotid-'. The 2-element Windkessel (△), 3-element Windkessel (×) and 1-D aortic (◯) algorithms were assessed in the 1-D (top), 'Aortic Coarctation' (second row), 'Normotensive' (third row), and 'Hypertensive' (bottom) datasets. y-axes are estimated minus reference cSBP values.

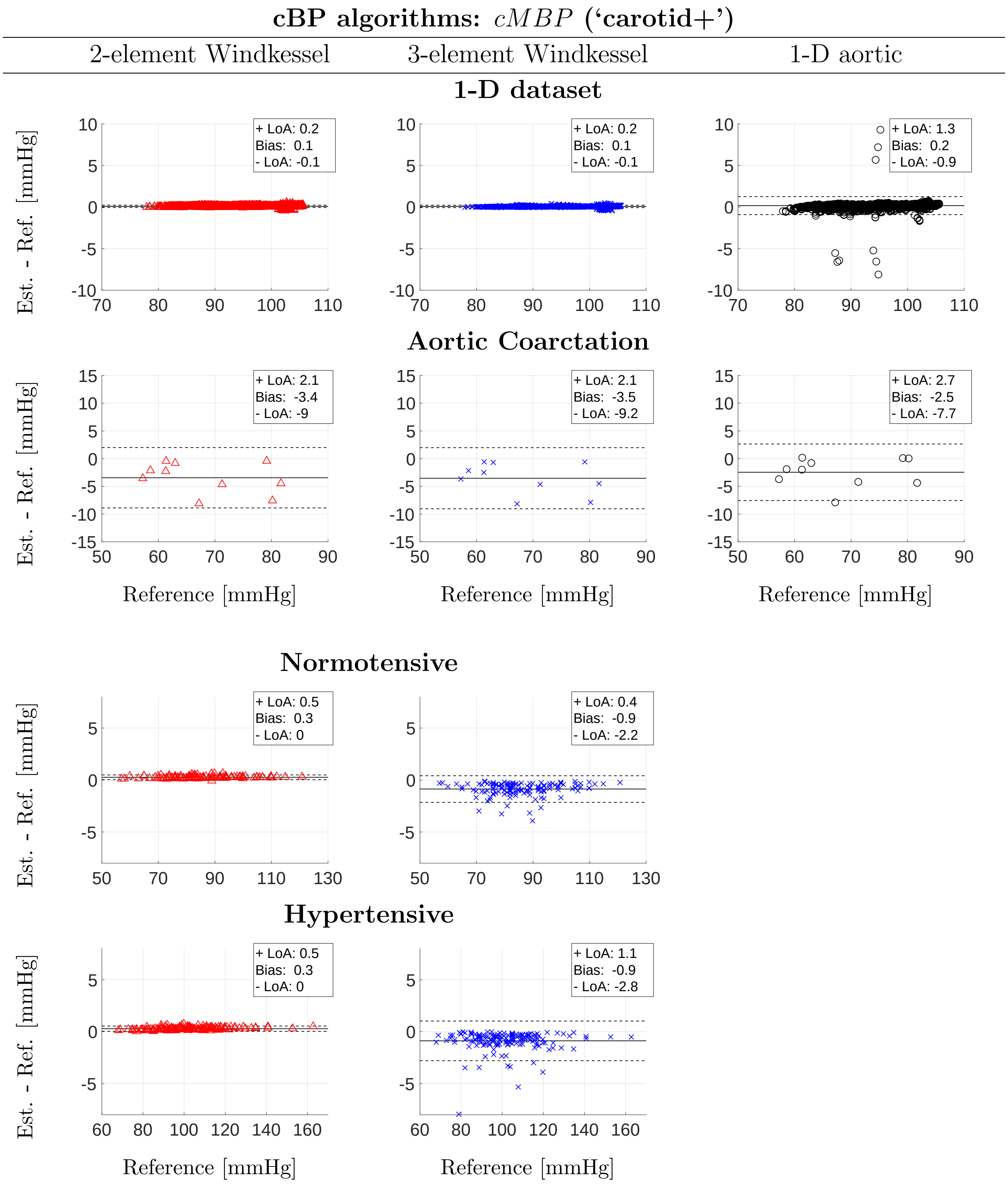

Figure S5: Bland-Altman plots for cMBP estimation for 'carotid+'. The 2-element Windkessel (△), 3-element Windkessel (×) and 1-D aortic (○) algorithms were assessed in the 1-D (top), 'Aortic Coarctation' (second row), 'Normotensive' (third row), and 'Hypertensive' (bottom) datasets. y-axes are estimated minus reference cMBP values.

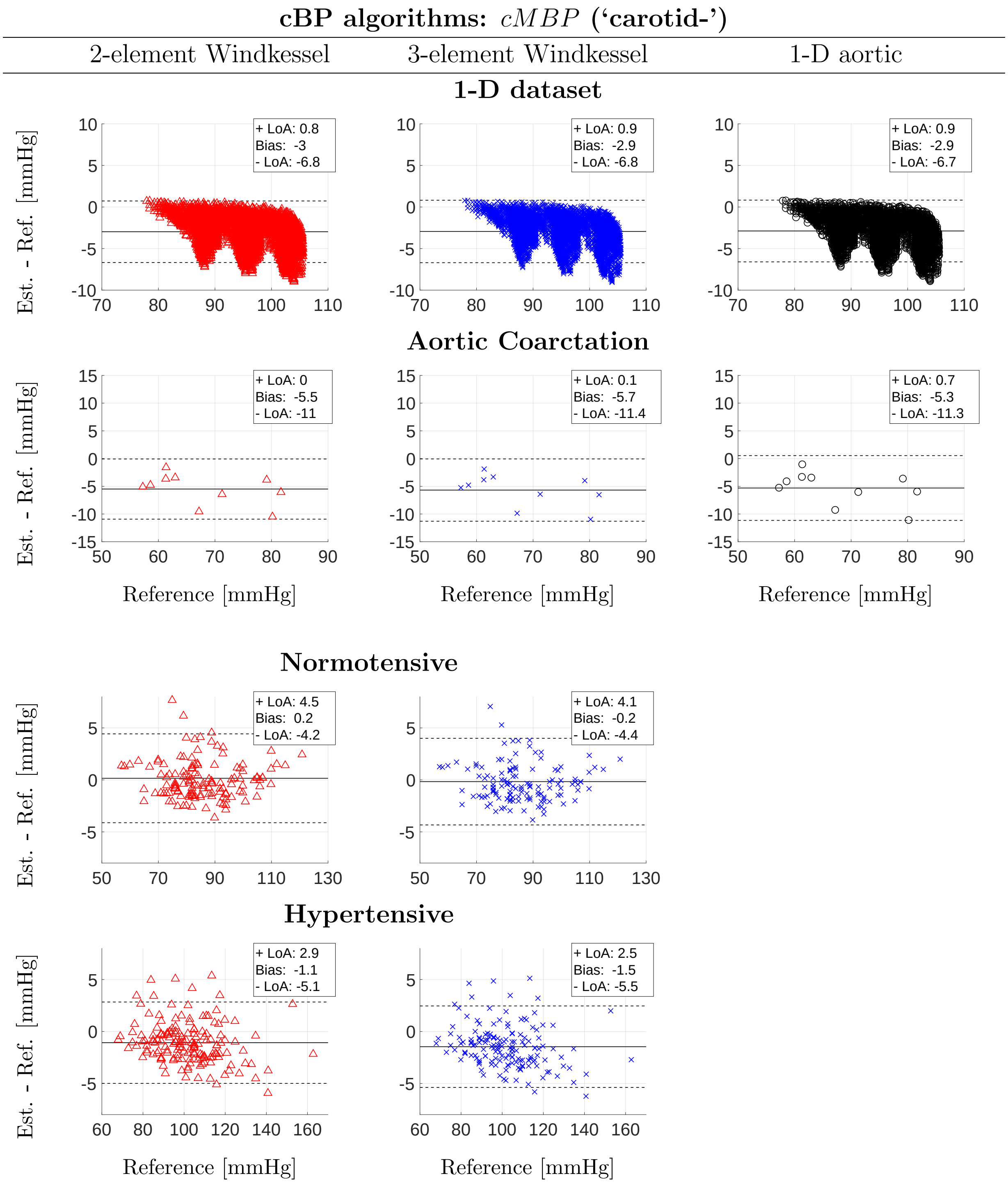

Figure S6: Bland-Altman plots for cMBP estimation for 'carotid-'. The 2-element Windkessel (△), 3-element Windkessel (×) and 1-D aortic (◯) algorithms were assessed in the 1-D (top), 'Aortic Coarctation' (second row), 'Normotensive' (third row), and 'Hypertensive' (bottom) datasets. y-axes are estimated minus reference cMBP values.

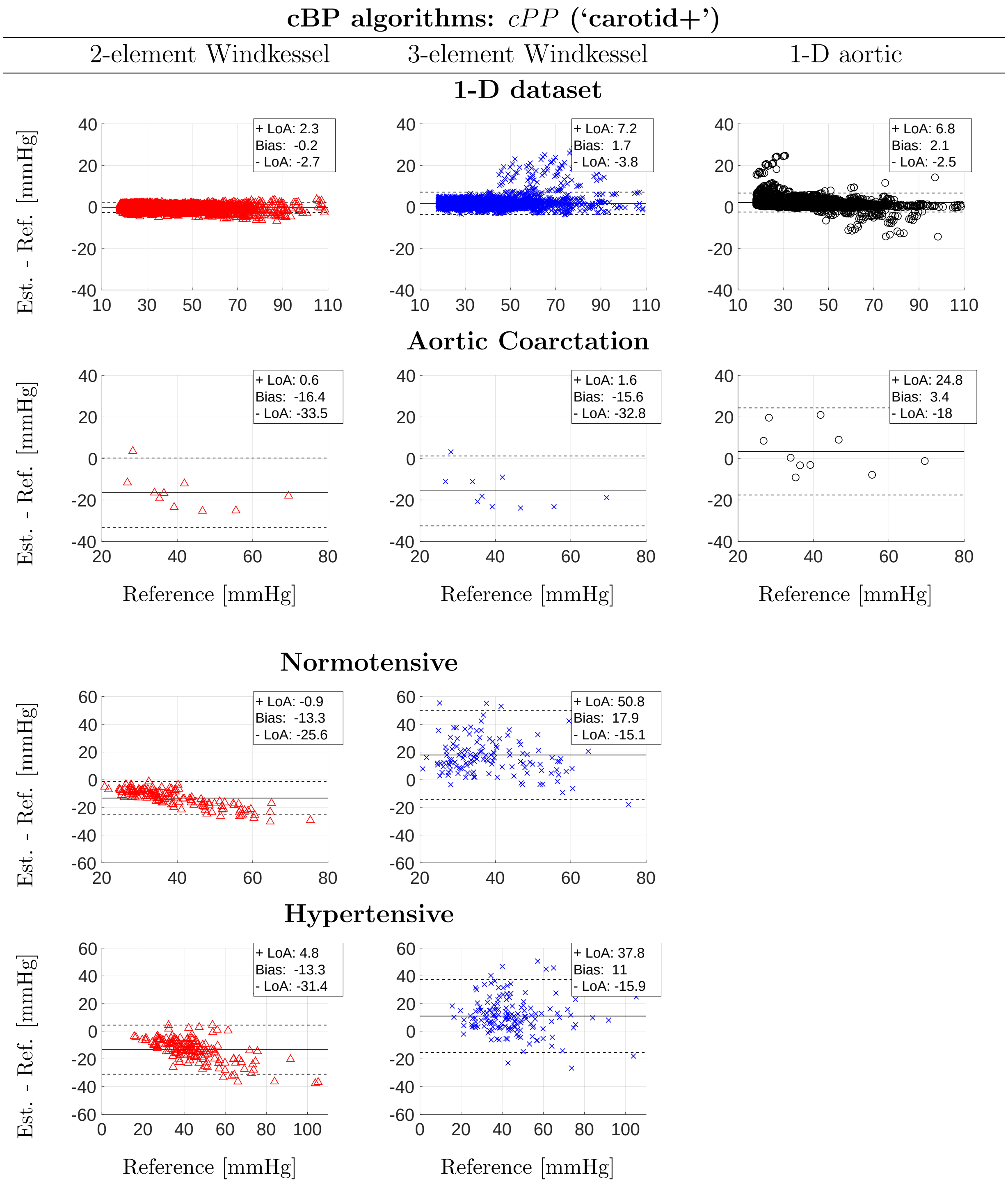

Figure S7: Bland-Altman plots for cPP estimation for 'carotid+'. The 2-element Windkessel (△), 3-element Windkessel (×) and '1D-Ao' (◯) algorithms were assessed in the 1-D (top), 'Aortic Coarctation' (second row), 'Normotensive' (third row), and 'Hypertensive' (bottom) datasets. y-axes are estimated minus reference cPP values.

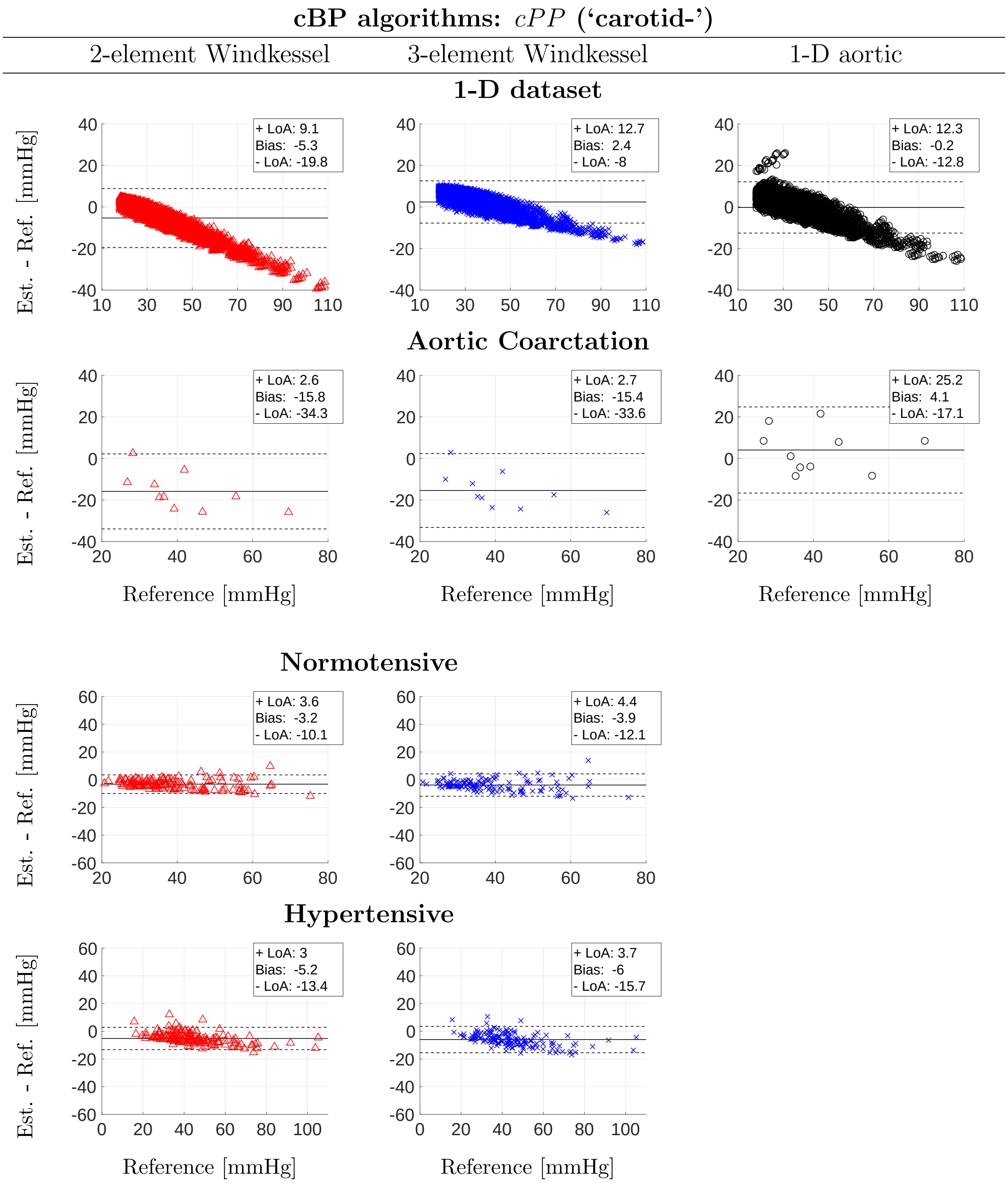

Figure S8: Bland-Altman plots for cPP estimation for 'carotid−'. The 2-element Windkessel (△), 3-element Windkessel (×) and '1D-Ao' (◯) algorithms were assessed in the 1-D (top), 'Aortic Coarctation' (second row), 'Normotensive' (third row), and 'Hypertensive' (bottom) datasets. y-axes are estimated minus reference cPP values.

3. Reference vs estimated cBP waves

3.1. cBP estimations in a set of randomly chosen 1-D model subjects

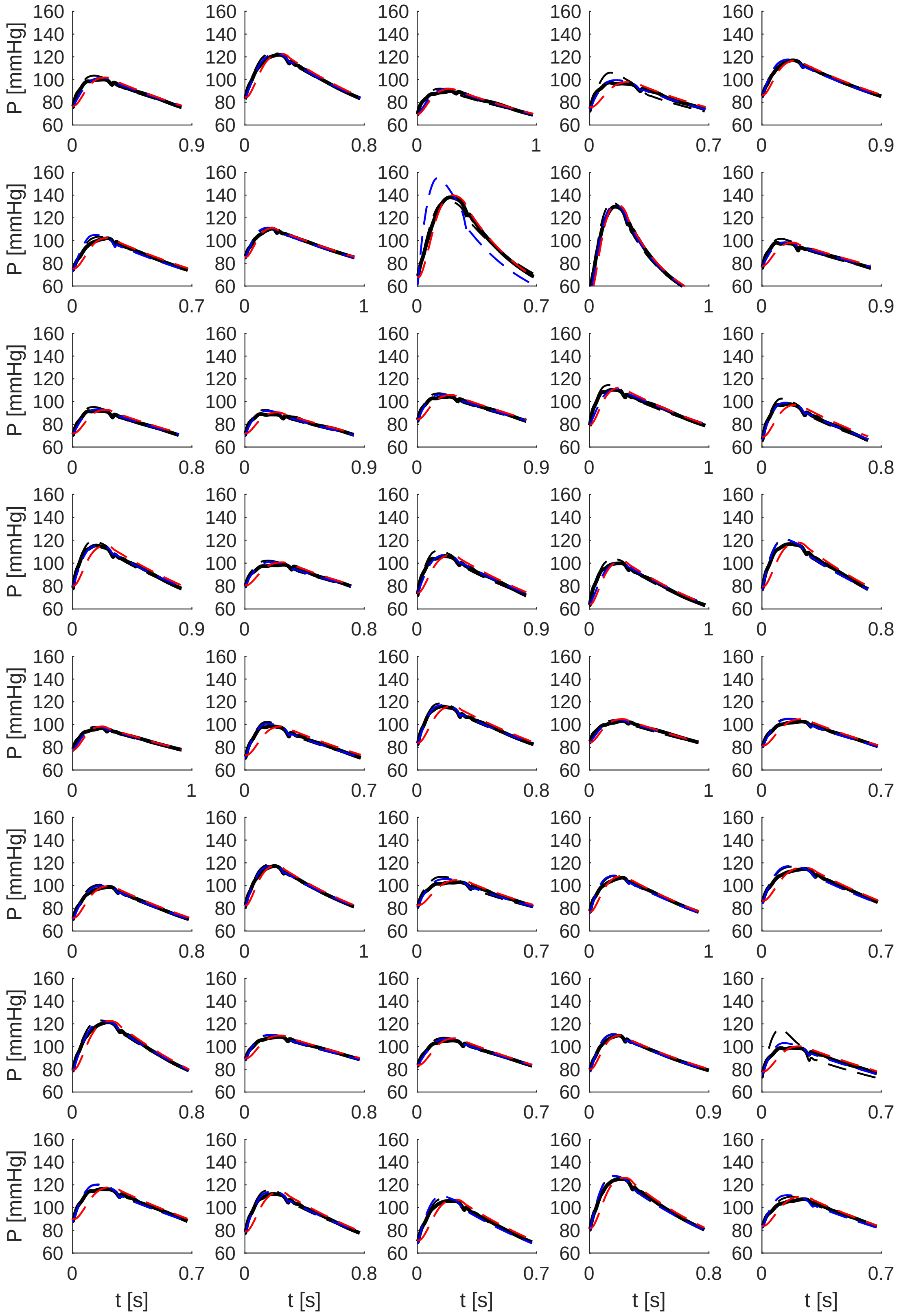

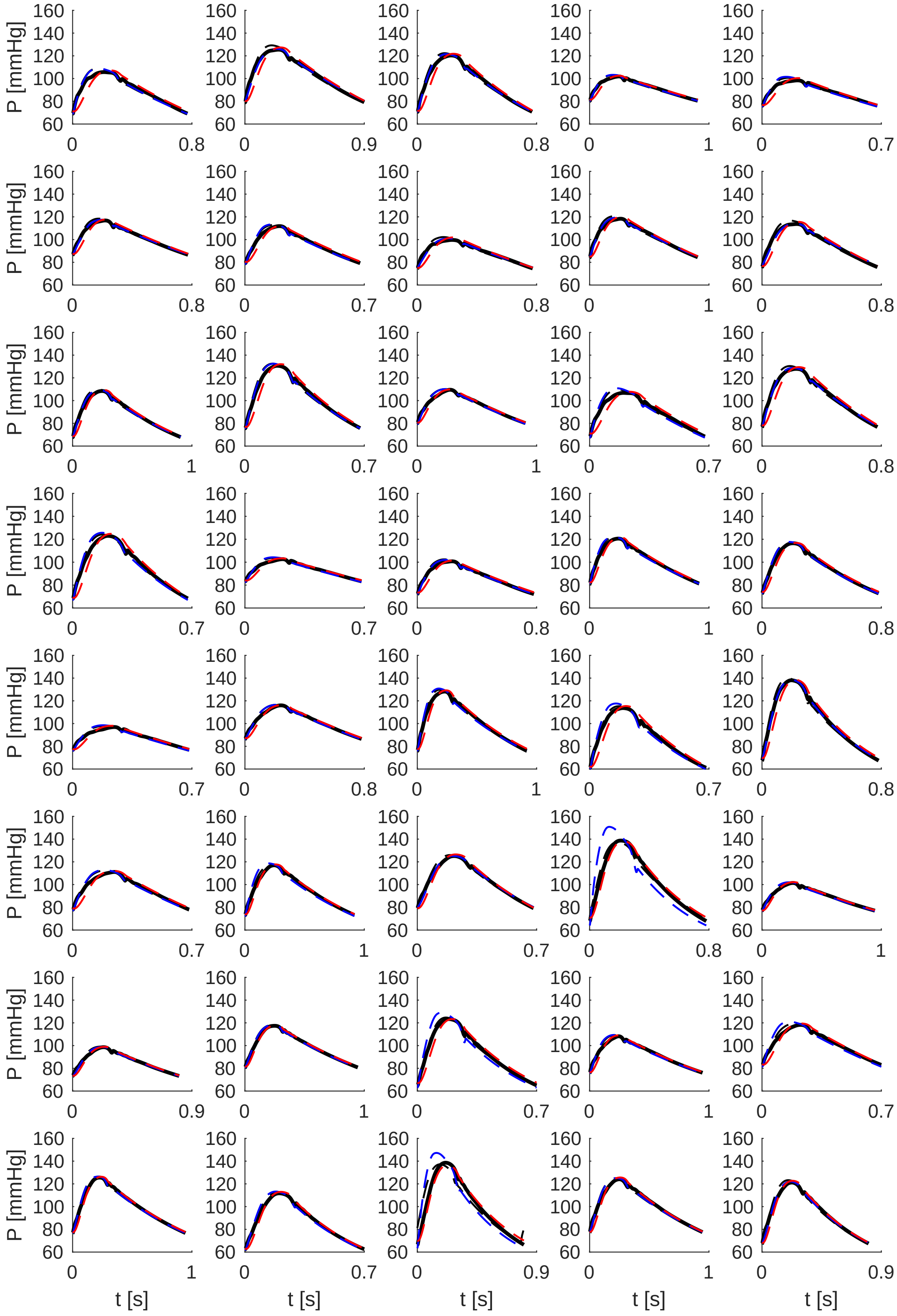

Figure S9: cBP wave estimations for 'carotid+'. cBP waves calculated using the 2-element Windkessel (red lines), 3-element Windkessel (blue lines), and 1-D aortic (black lines) cBP algorithms. They are compared against reference cBP waves from the 1-D model dataset (thick black lines).

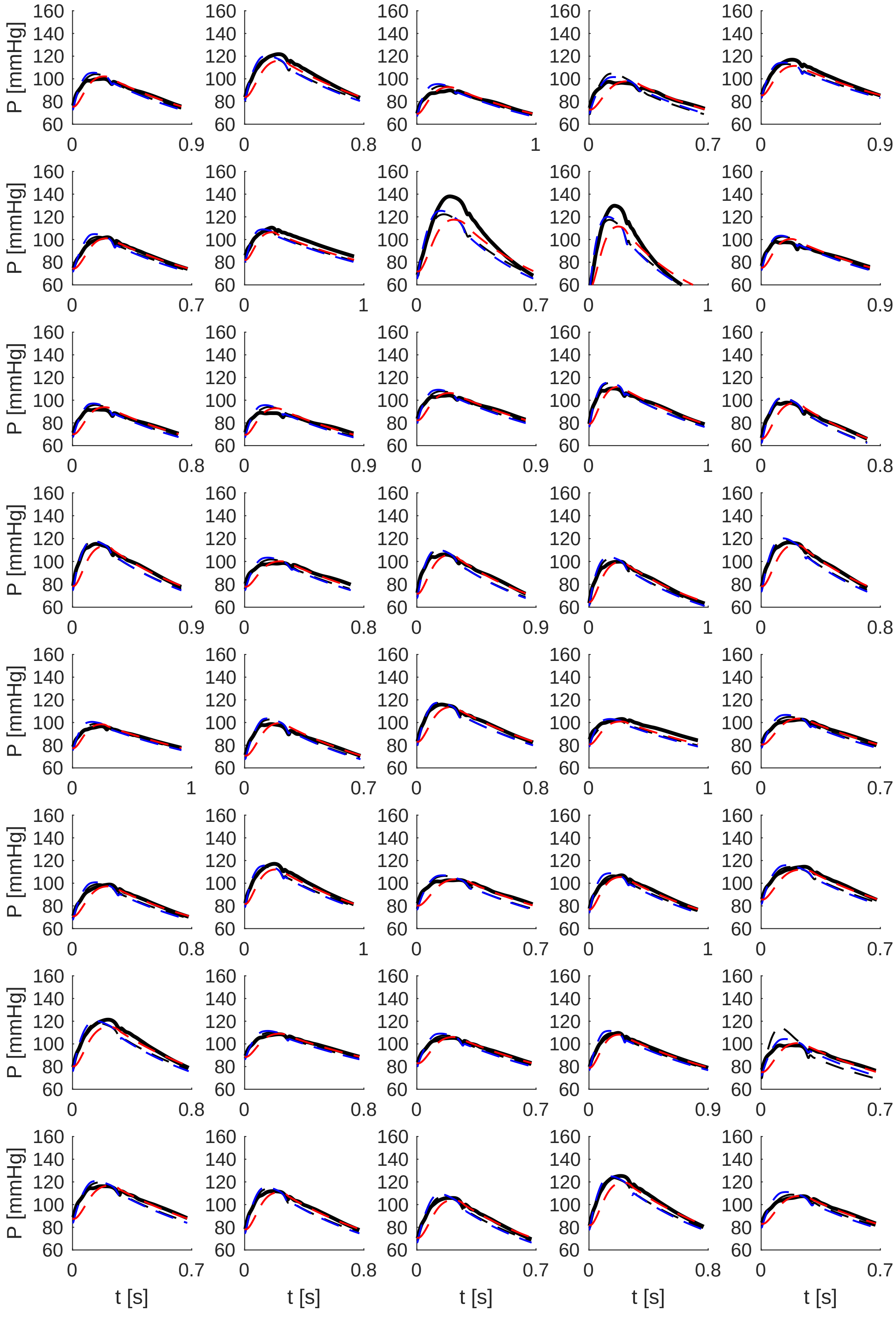

P [mmHg]
160
140
120
100
80
60
0
0.9
0.8
1
0.7
t [s]

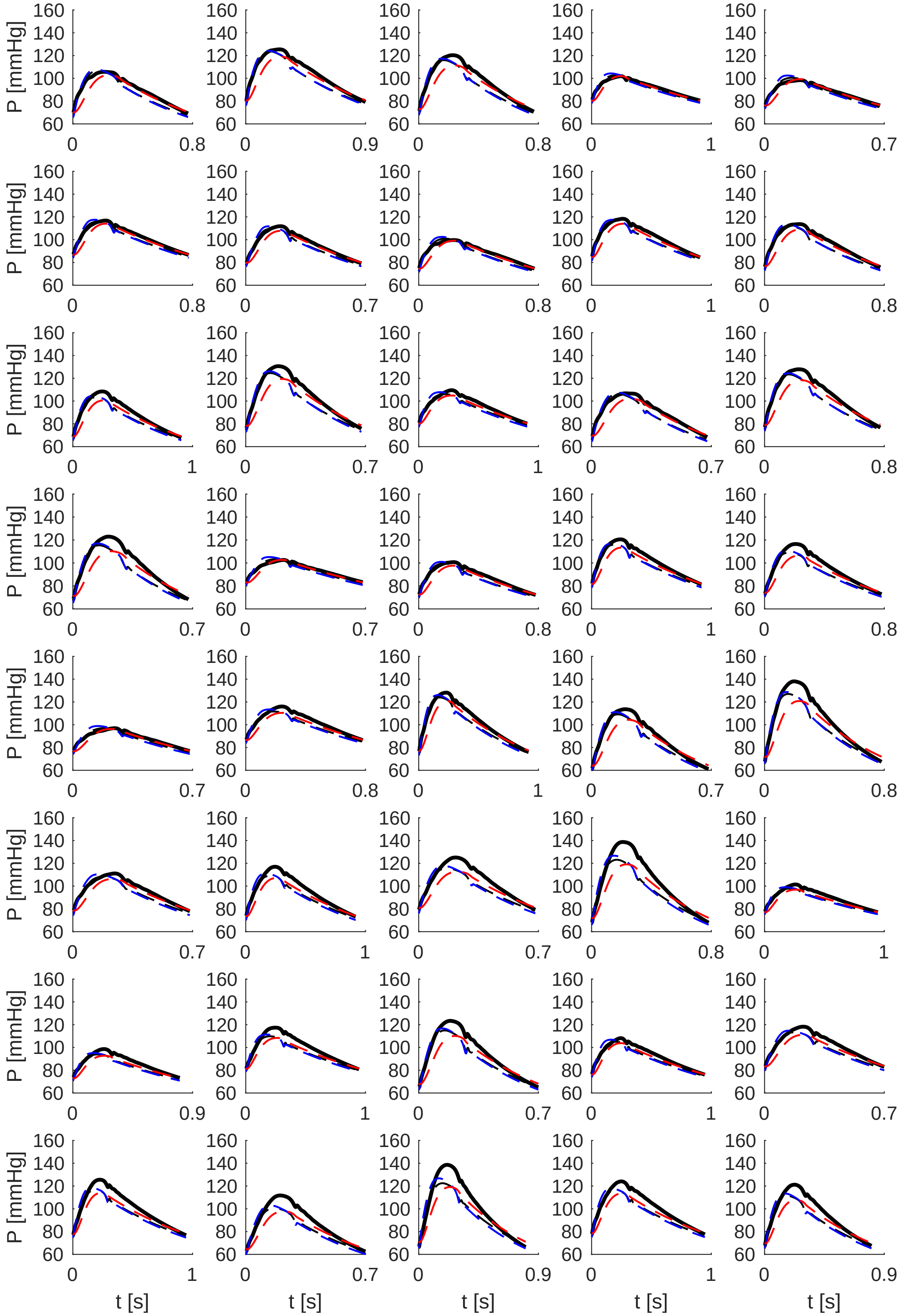

Figure S10: cBP wave estimations for ‘carotid-’. cBP waves calculated using the 2-element Windkessel (red lines), 3-element Windkessel (blue lines), and 1-D aortic (black lines) cBP algorithms. They are compared against reference cBP waves from the 1-D model dataset (thick black lines).

3.2. cBP estimations in the 'Aortic Coarctation' dataset

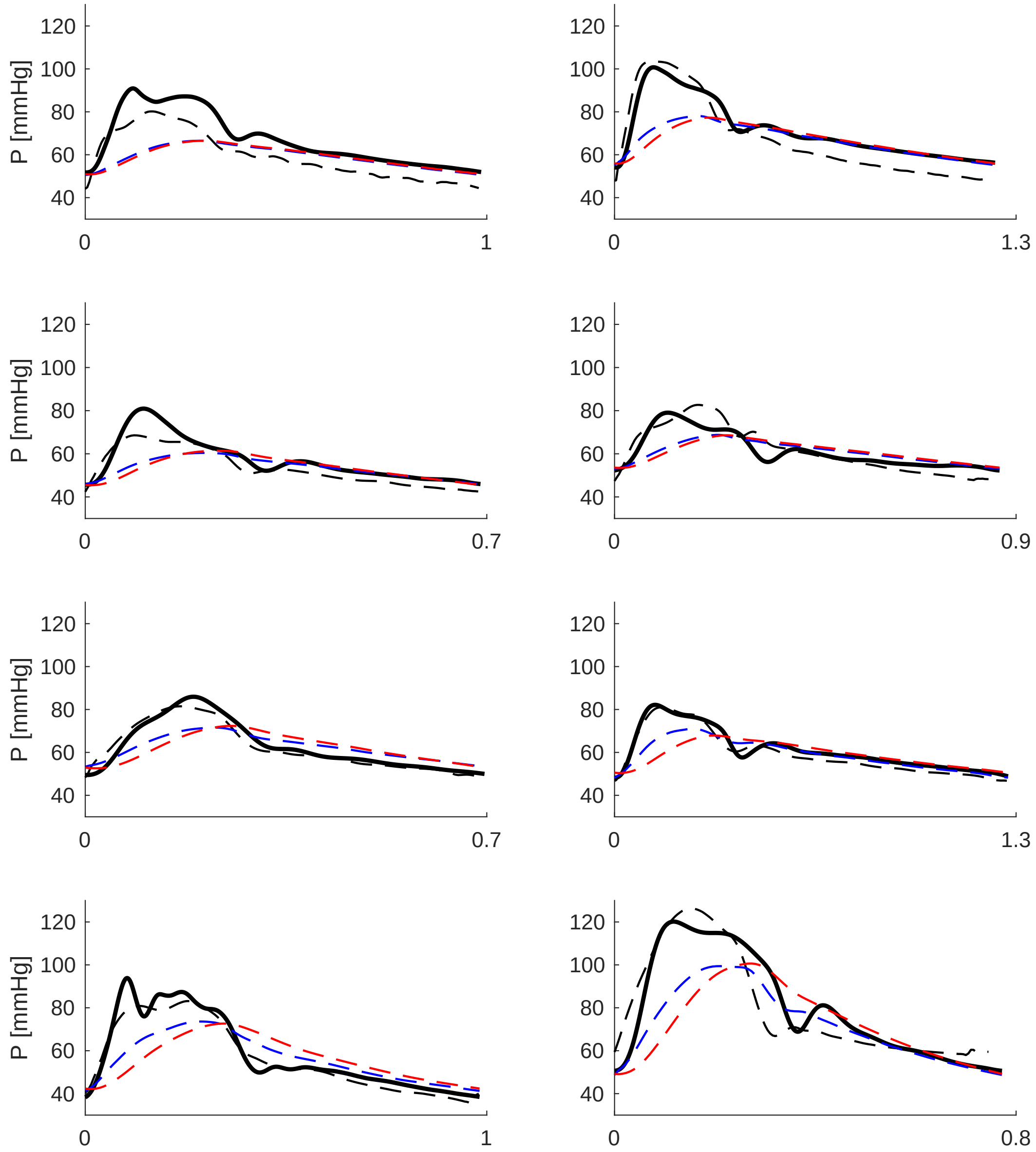

Figure S11: cBP wave estimations for 'carotid+'. cBP waves calculated using the 2-element Windkessel (red lines), 3-element Windkessel (blue lines), and 1-D aortic (black lines) cBP algorithms. They are compared against catheter measurements from the 'Aortic Coarctation' dataset (thick black lines).

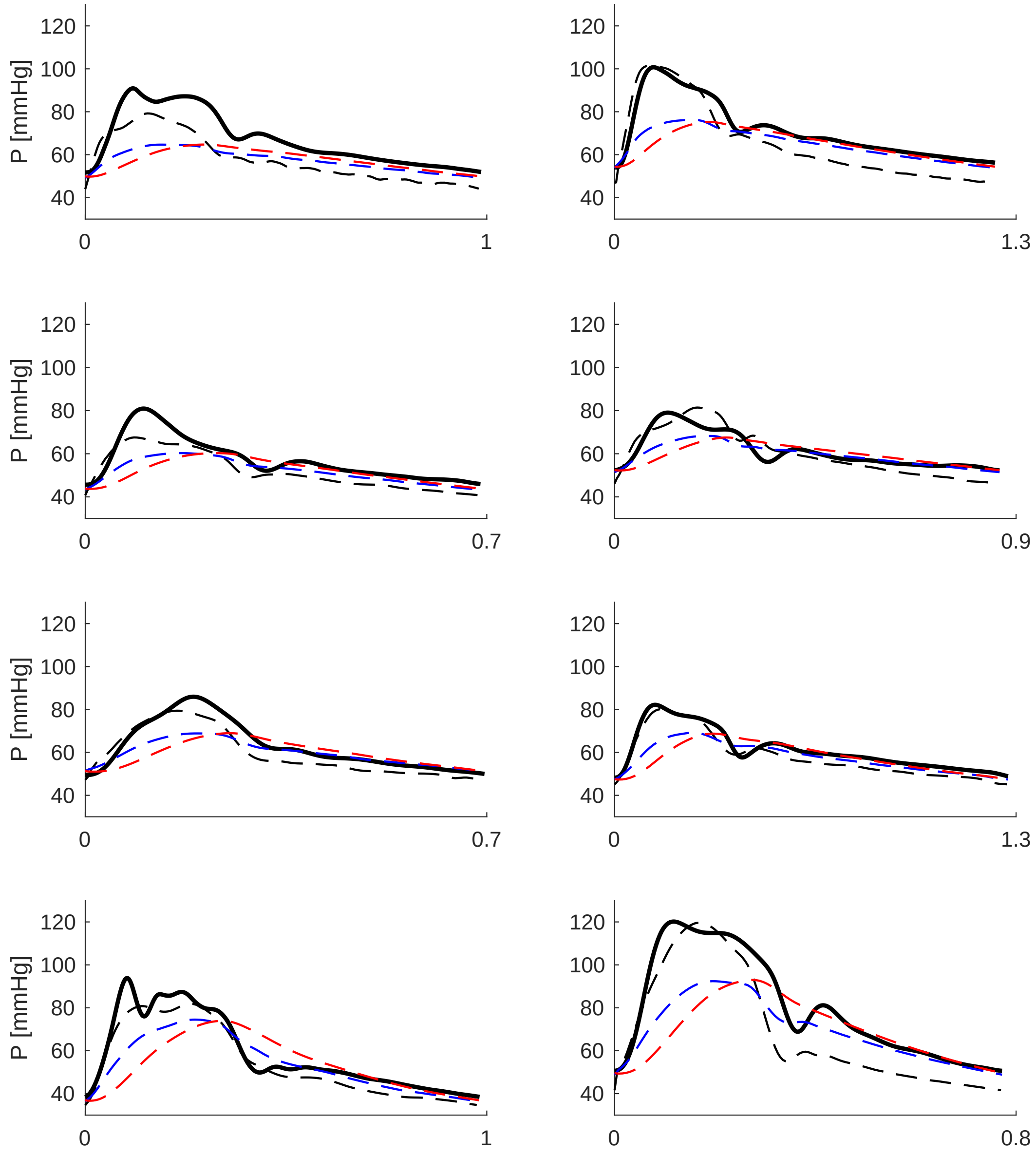

Figure S12: cBP wave estimations for 'carotid-'. cBP waves calculated using the 2-element Windkessel (red lines), 3-element Windkessel (blue lines), and 1-D aortic (black lines) cBP algorithms. They are compared against catheter measurements from the 'Aortic Coarctation' dataset (thick black lines).

3.3. cBP estimations in the 'Normotensive' dataset

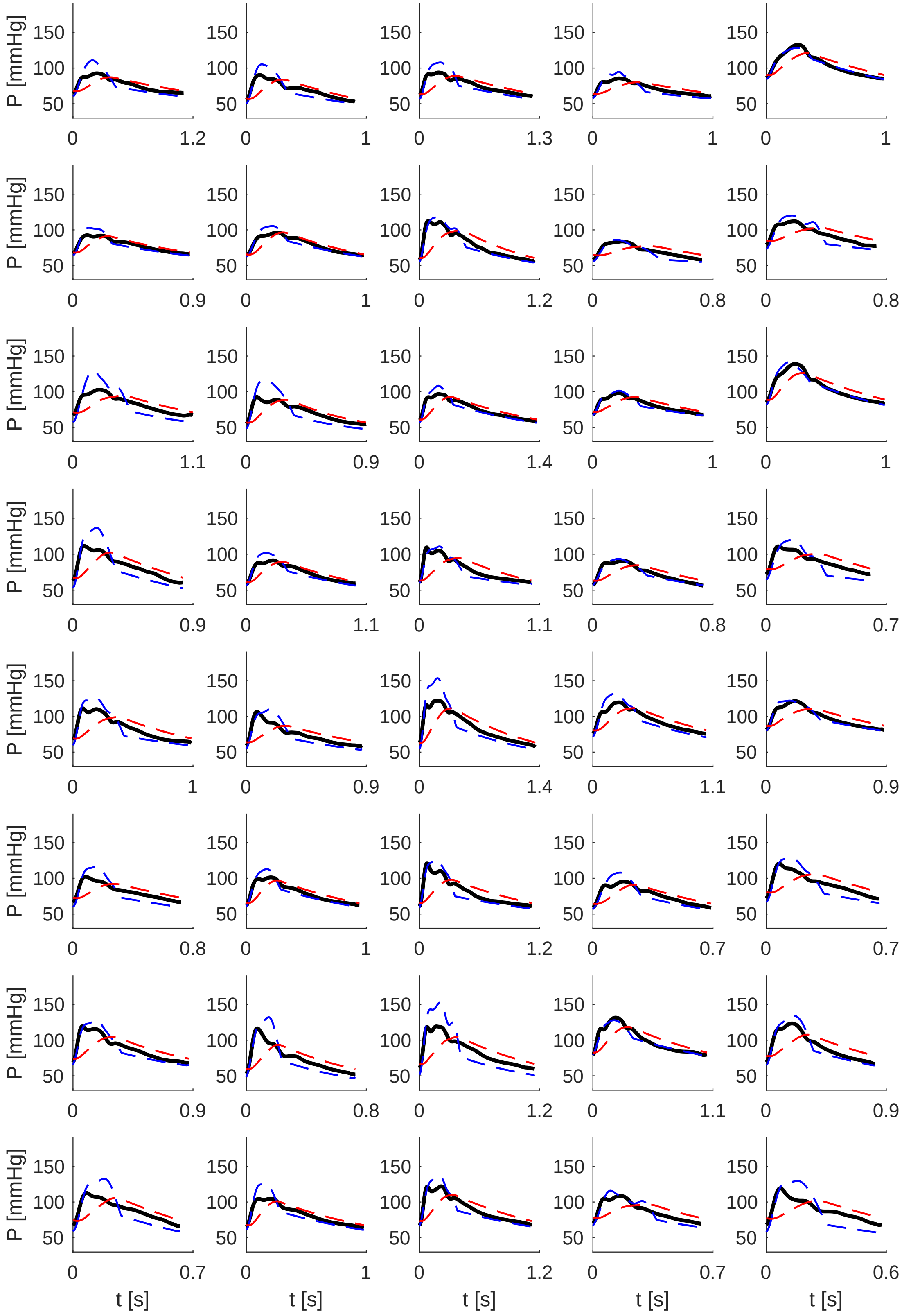

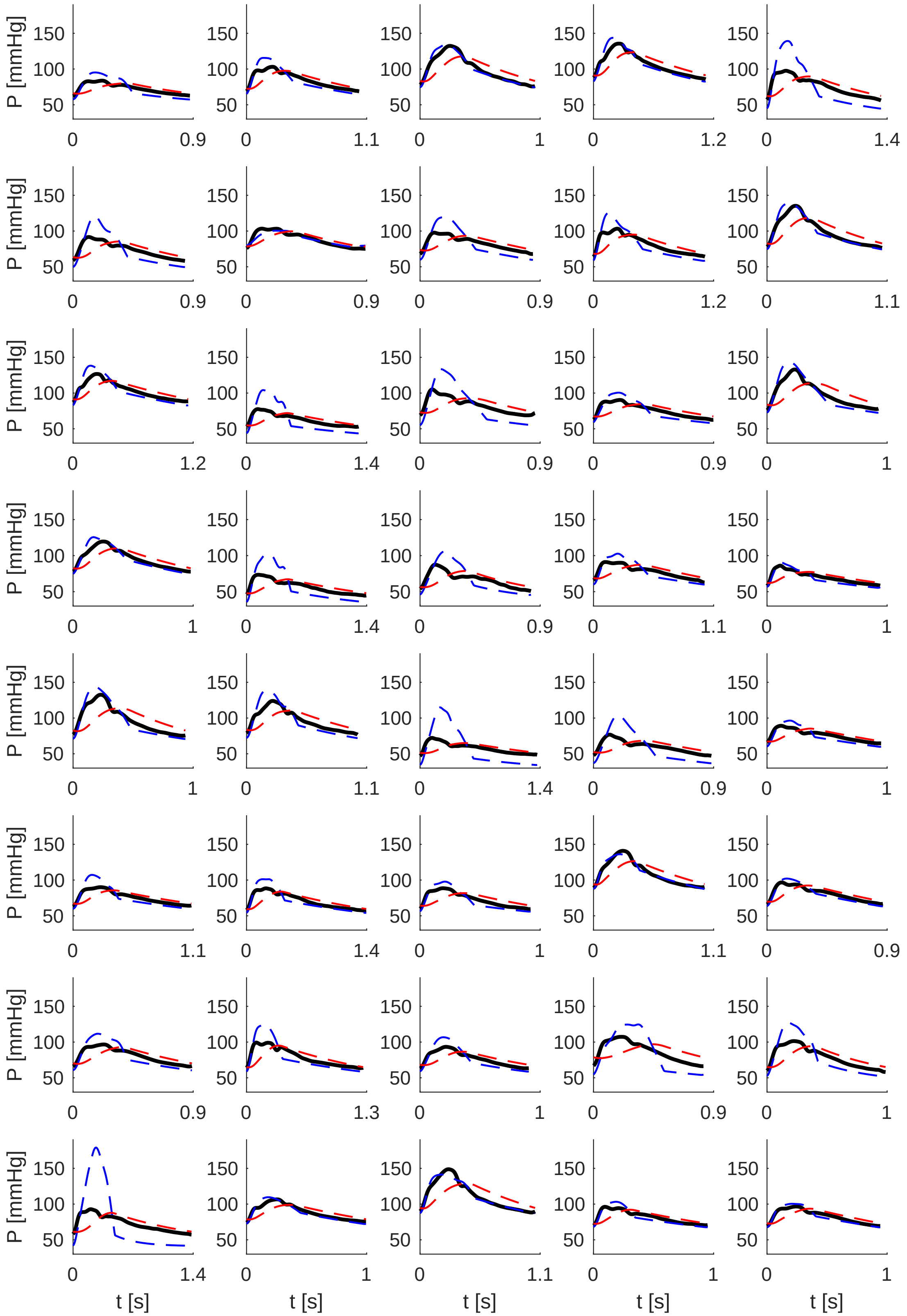
P [mmHg]
150
100
50
0
t [s]
0.9
1.1
1
1.2
1.4
1.3

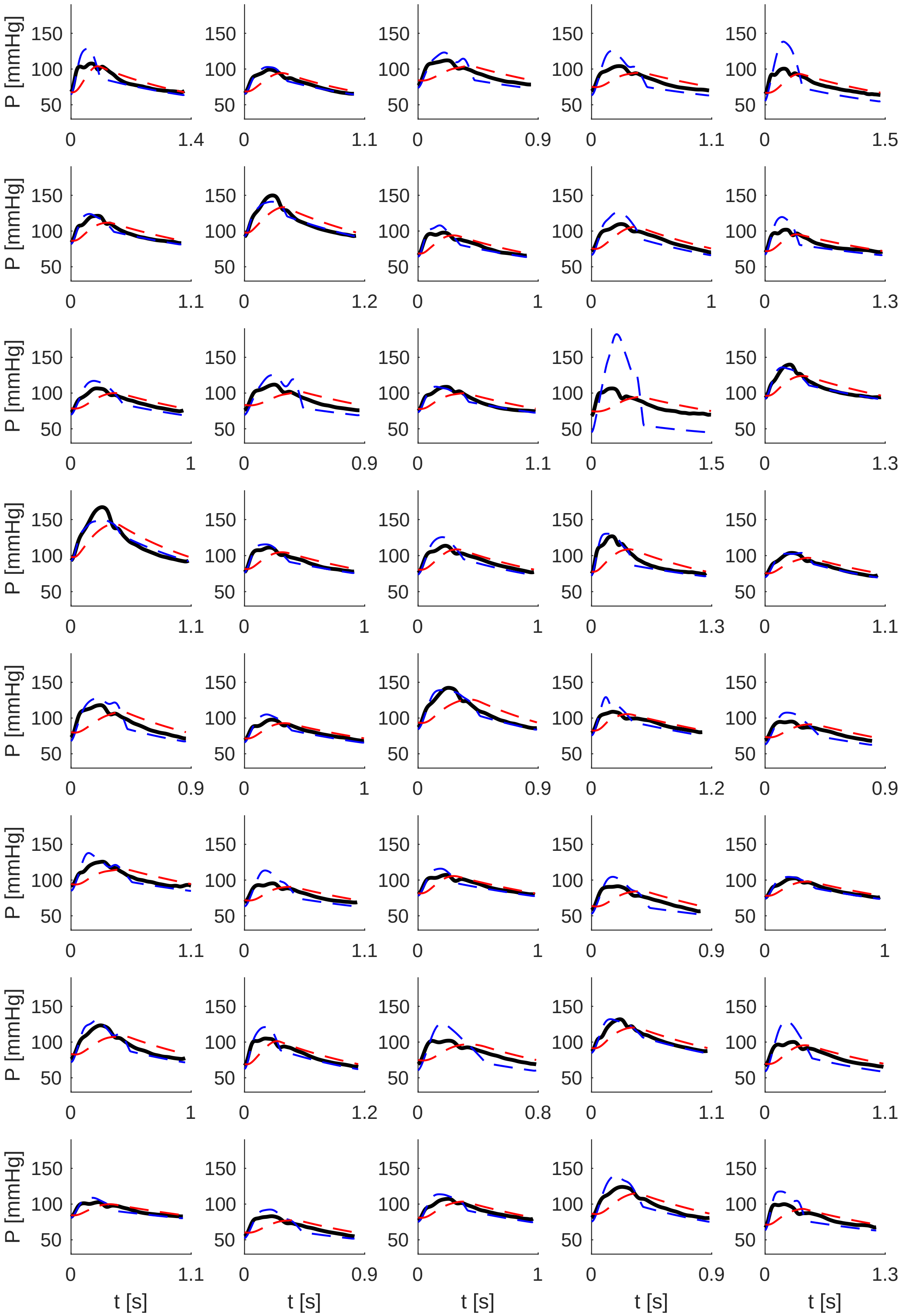
P [mmHg]
150
100
50
t [s]

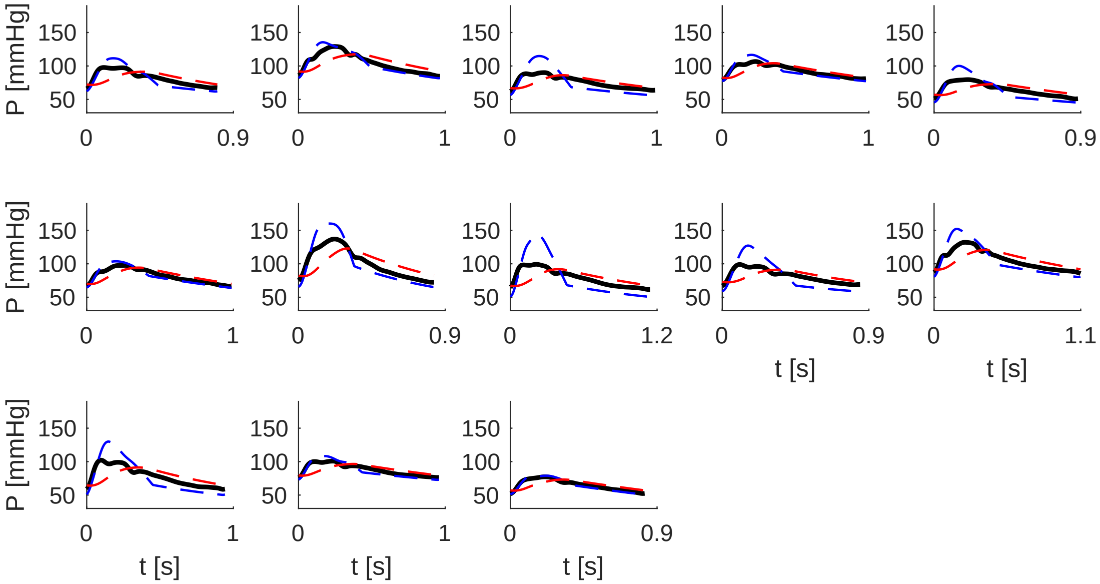

Figure S13: cBP wave estimations for 'carotid+'. cBP waves calculated using the 2-element Windkessel (red lines) and 3-element Windkessel (blue lines) cBP algorithms. They are compared against reference cBP waves from the 'Normotensive' dataset (thick black lines).

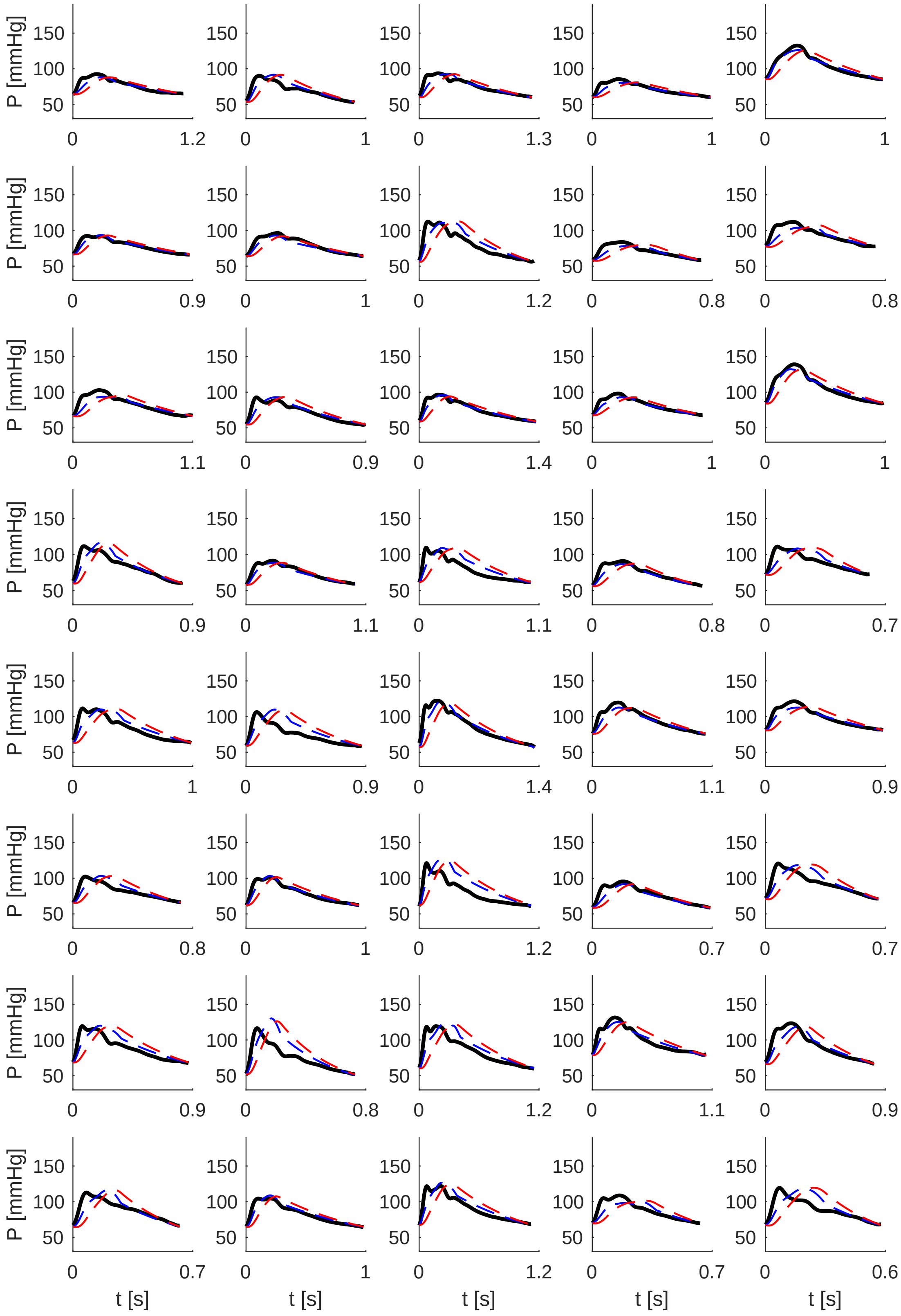
P [mmHg]
150
100
50
t [s]
0
1.2
1
1.3
0.9
0.8
1.1
1.4
0.7
0.6

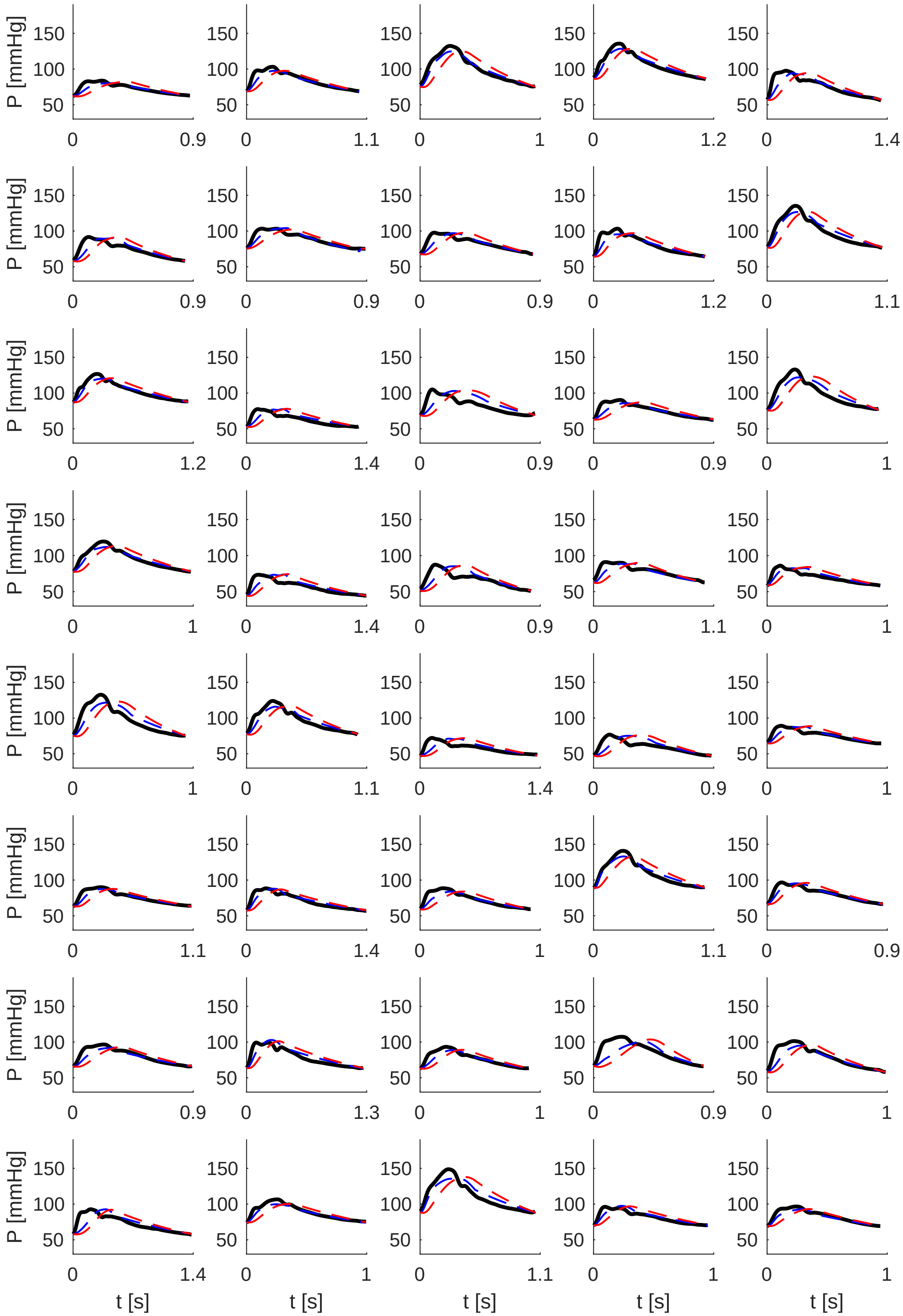
P [mmHg]
150
100
50
t [s]
0
0.9
1.1
1
1.2
1.4
1.3

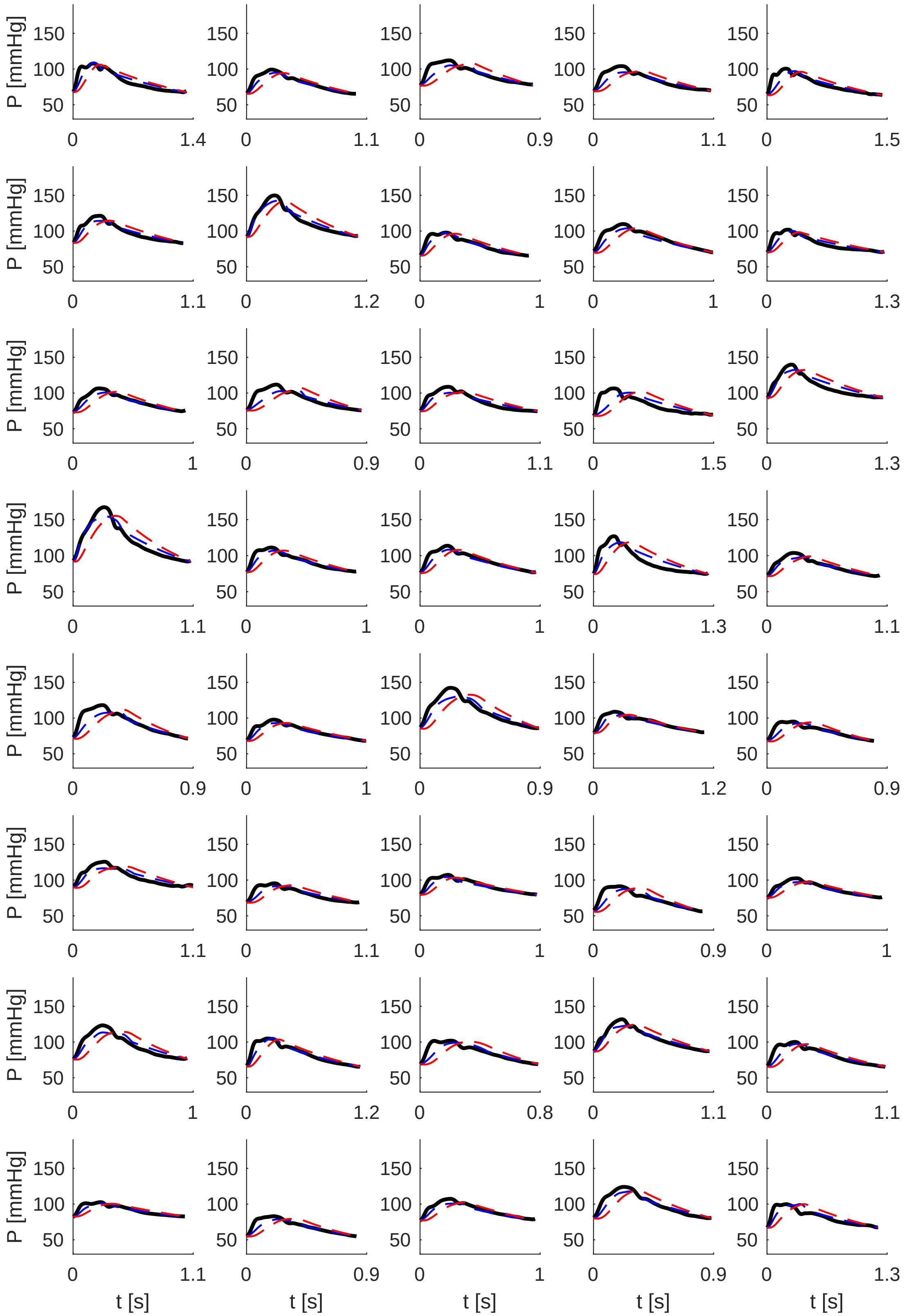
P [mmHg]
150
100
50
0
1.4
1.1
0.9
1.1
1.5
1.1
1.2
1
1
1.3
1
0.9
1.1
1.5
1.3
1.1
1
1
1.3
1.1
0.9
1
0.9
1.2
0.9
1.1
1.1
1
0.9
1
1
1.2
0.8
1.1
1.1
1.1
0.9
1
0.9
1.3
t [s]

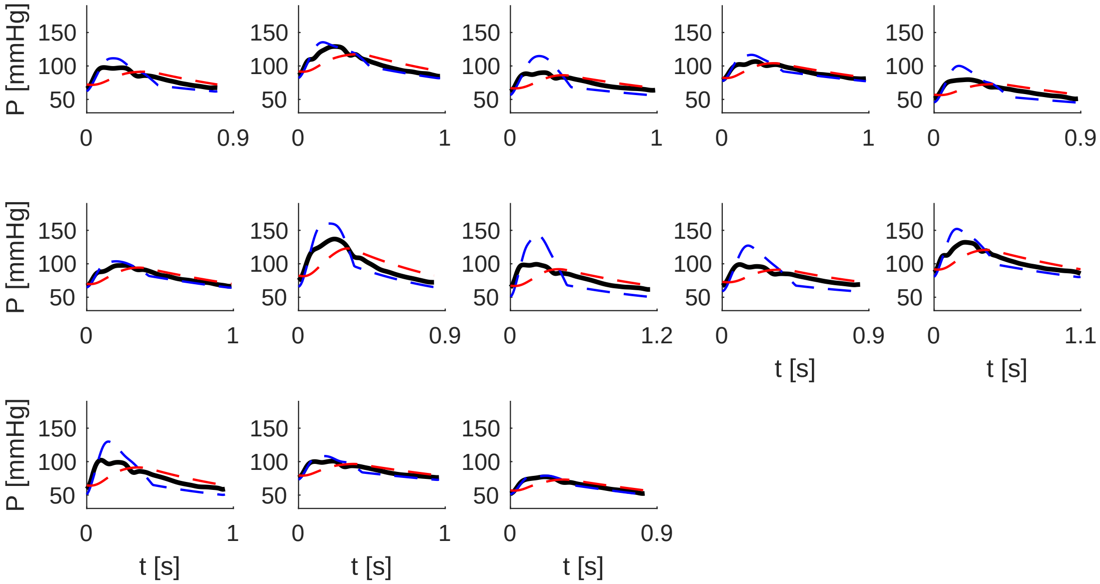

Figure S14: cBP wave estimations for 'carotid-'. cBP waves calculated using the 2-element Windkessel (red lines) and 3-element Windkessel (blue lines) cBP algorithms. They are compared against reference cBP waves from the 'Normotensive' dataset (thick black lines).

3.4. cBP estimations in the 'Hypertensive' dataset

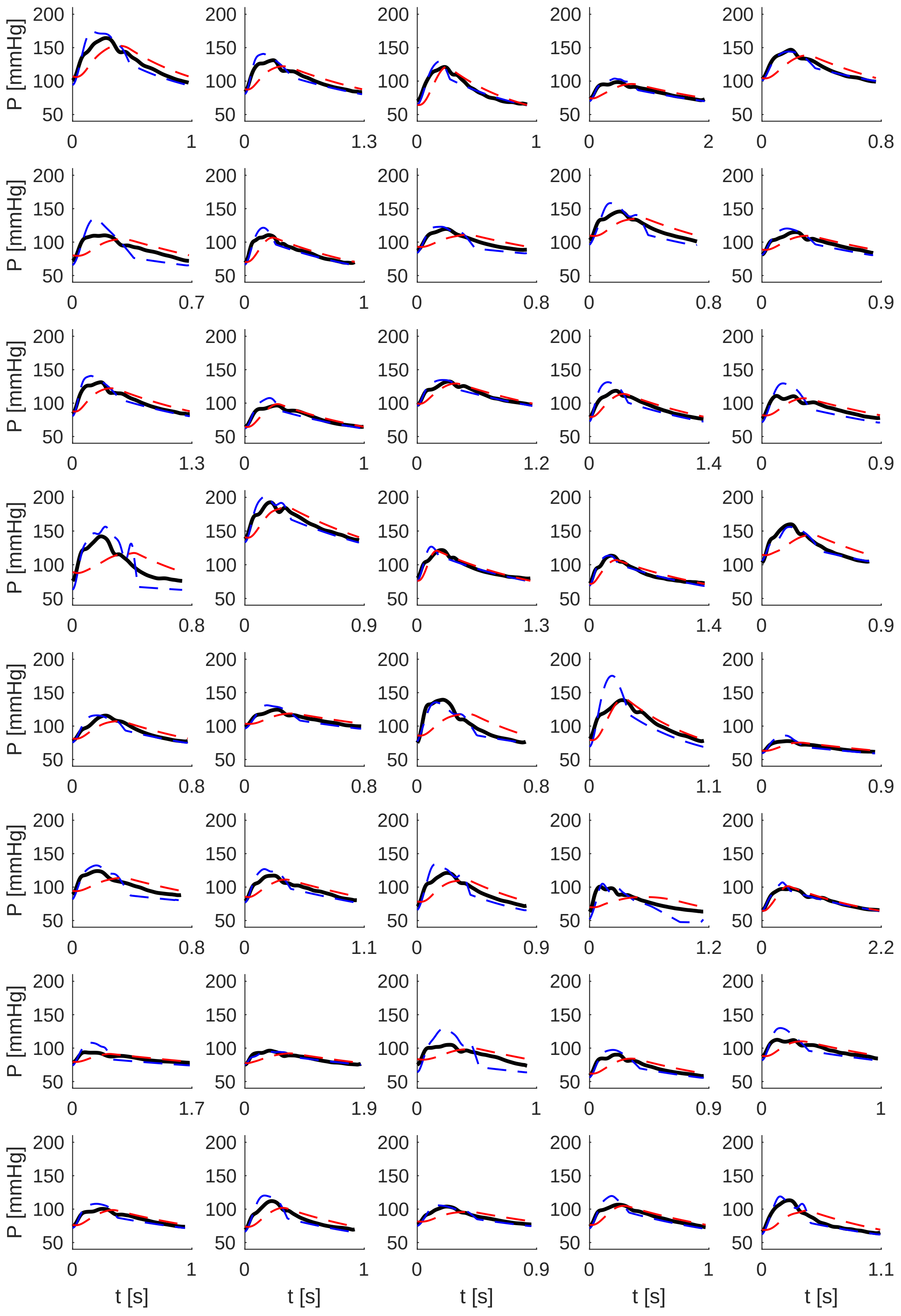

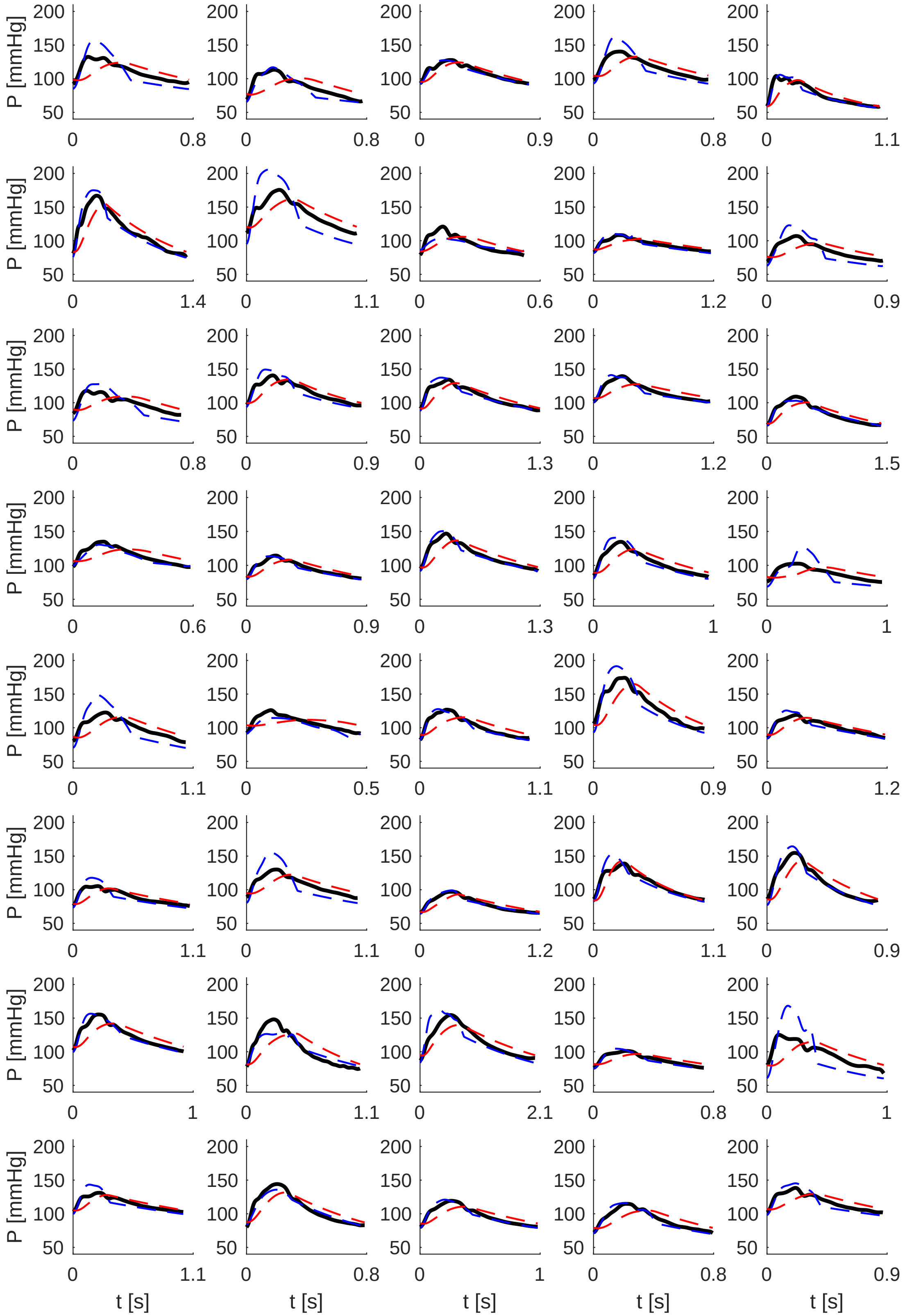
P [mmHg]
200
150
100
50
0
0.8
1.1
0.9
1.4
0.6
1.2
1.3
1.5
1
0.5
2.1
t [s]

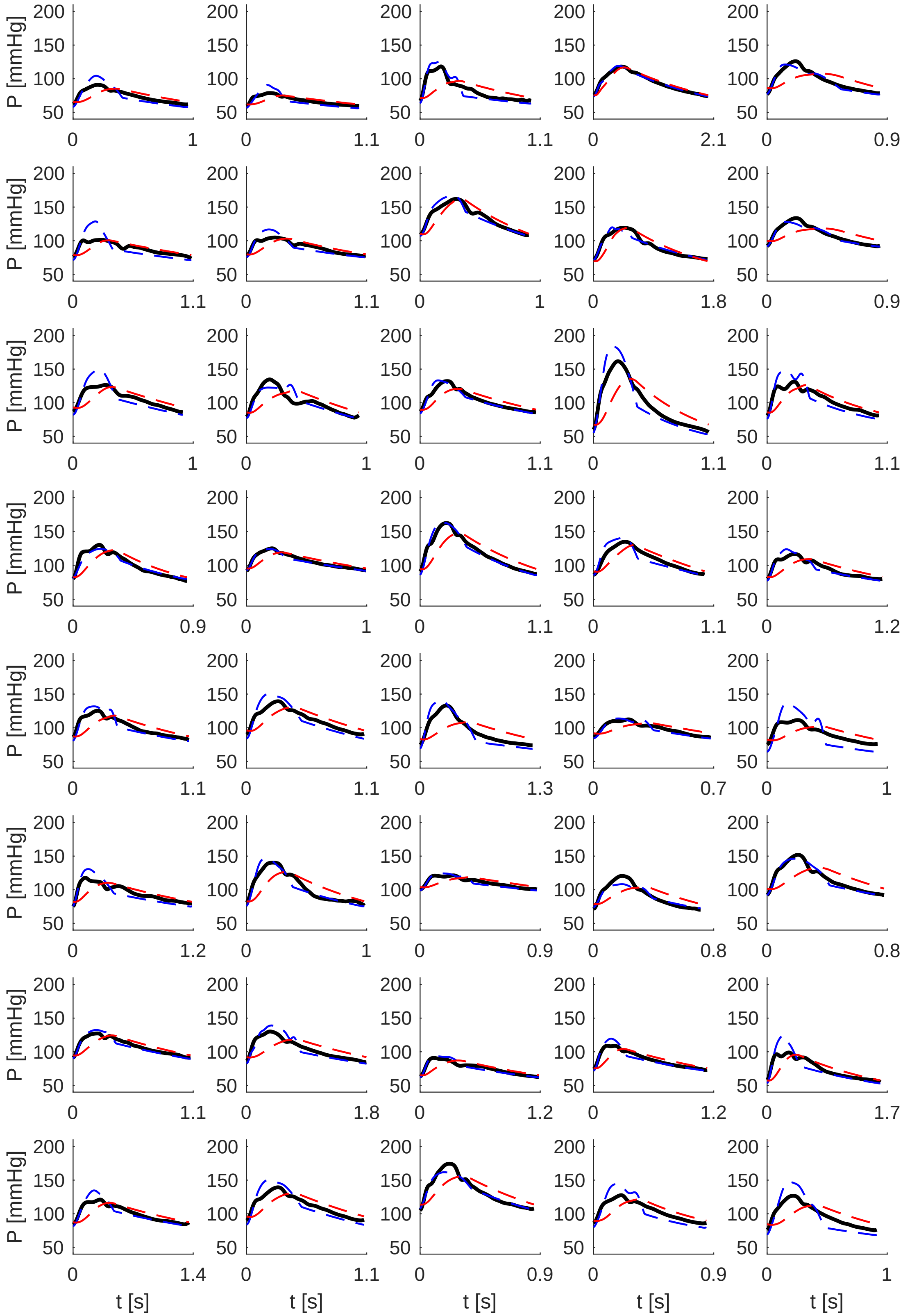
P [mmHg]
200
150
100
50
0
1
1.1
1.1
2.1
0.9
1.1
1.1
1
1.8
0.9
1
1
1.1
1.1
1.1
0.9
1
1.1
1.1
1.2
1.1
1.1
1.3
0.7
1
1.2
1
0.9
0.8
0.8
1.1
1.8
1.2
1.2
1.7
1.4
1.1
0.9
0.9
1
t [s]

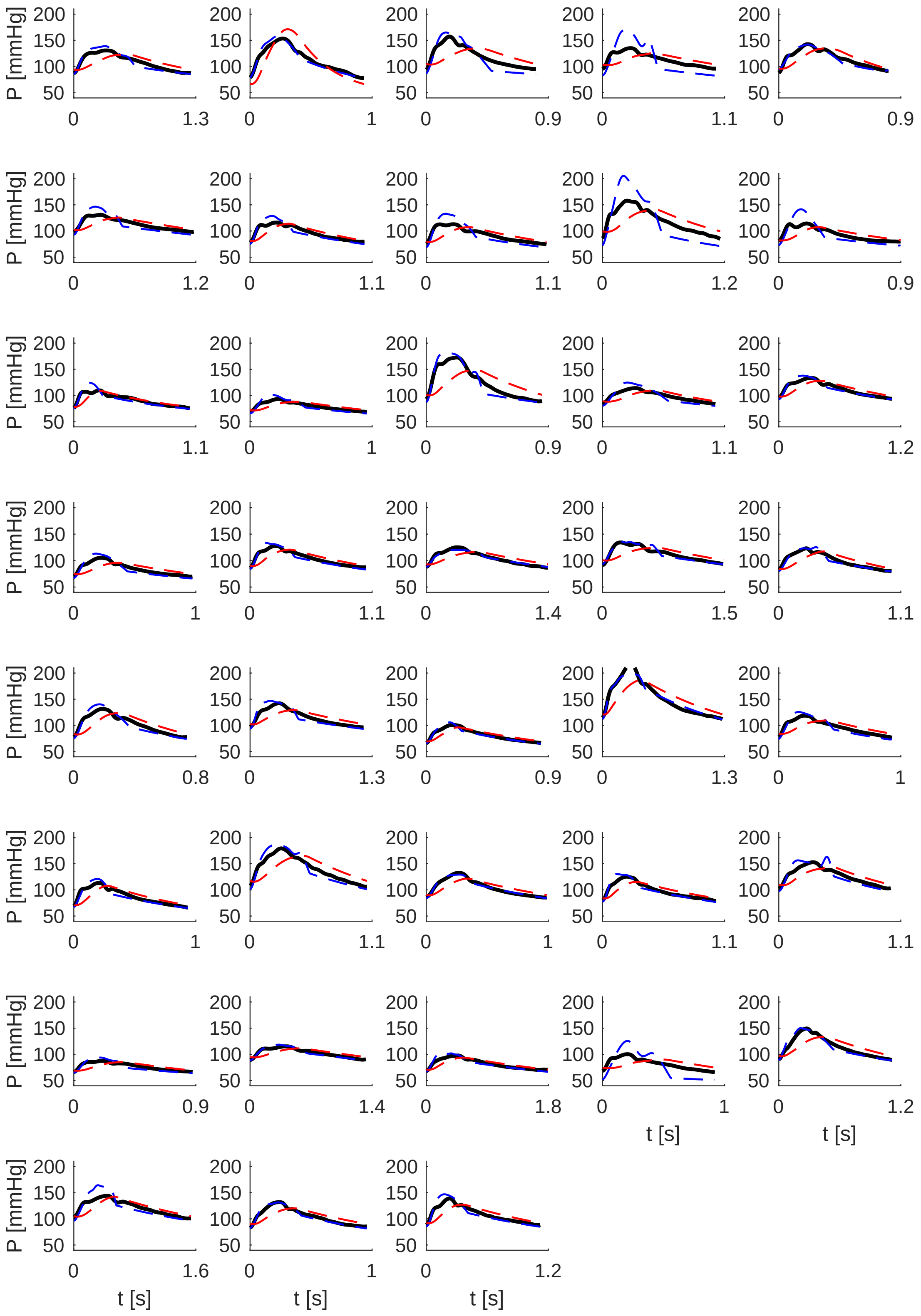

Figure S15: cBP wave estimations for 'carotid+'. cBP waves calculated using the 2-element Windkessel (red lines) and 3-element Windkessel (blue lines) cBP algorithms. They are compared against reference cBP waves from the 'Hypertensive' dataset (thick black lines).

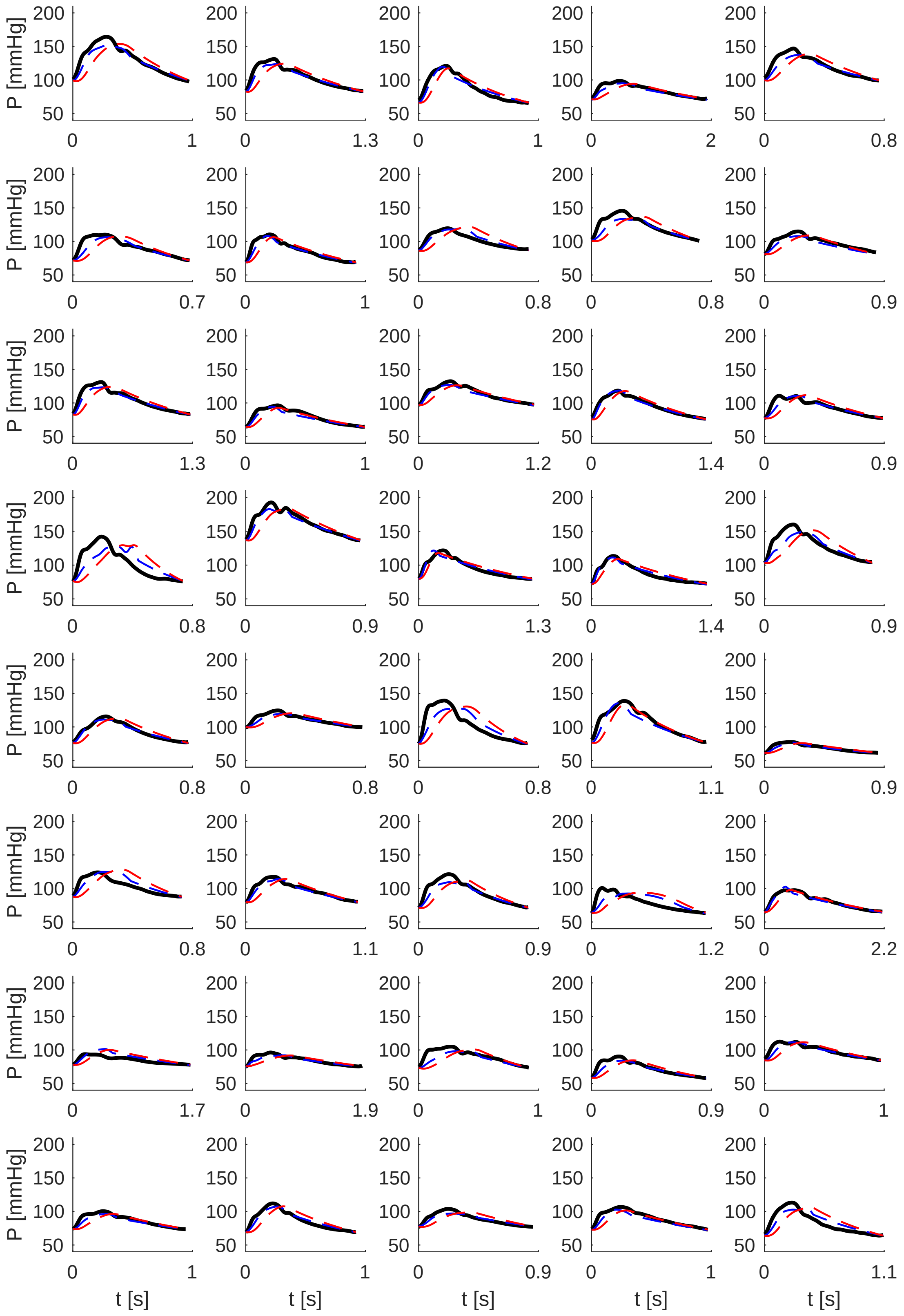
P [mmHg]
t [s]
200
150
100
50

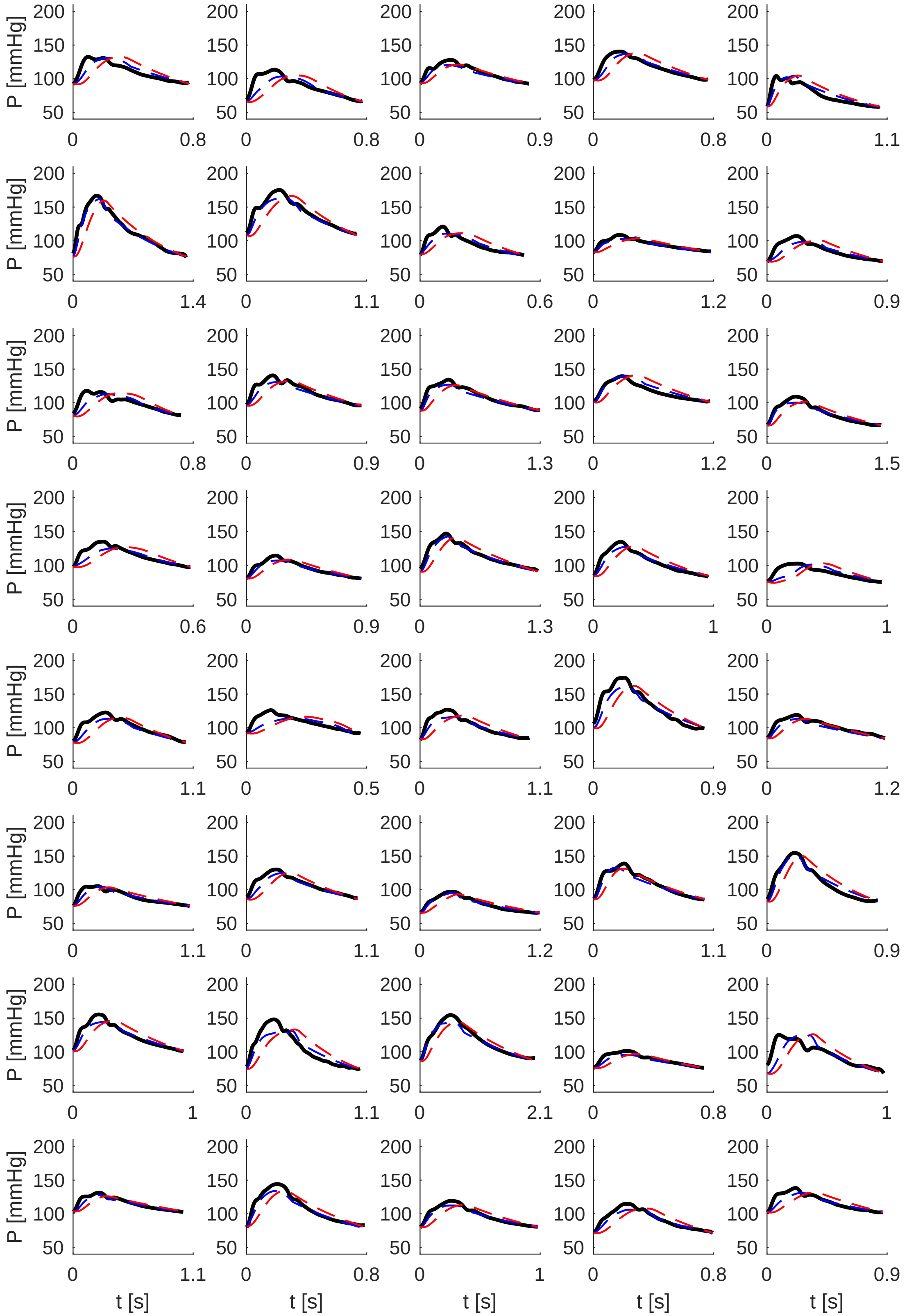
P [mmHg]
200
150
100
50
t [s]
0
0.8
0.8
0.9
0.8
1.1
1.4
1.1
0.6
1.2
0.9
0.8
0.9
1.3
1.2
1.5
0.6
0.9
1.3
1
1
1.1
0.5
1.1
0.9
1.2
1.1
1.1
1.2
1.1
0.9
1
1.1
2.1
0.8
1
1.1
0.8
1
0.8
0.9

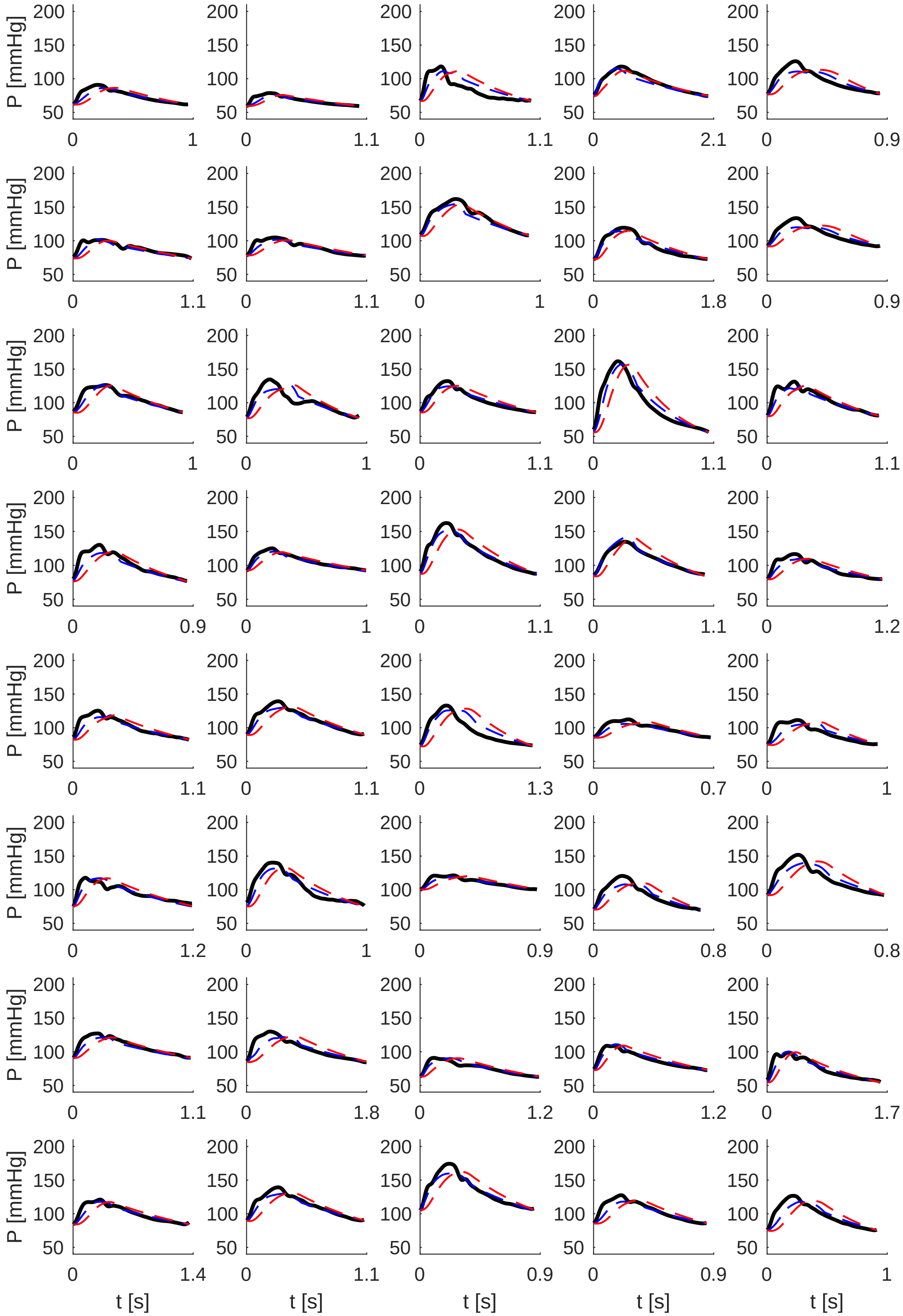
P [mmHg]
200
150
100
50
t [s]

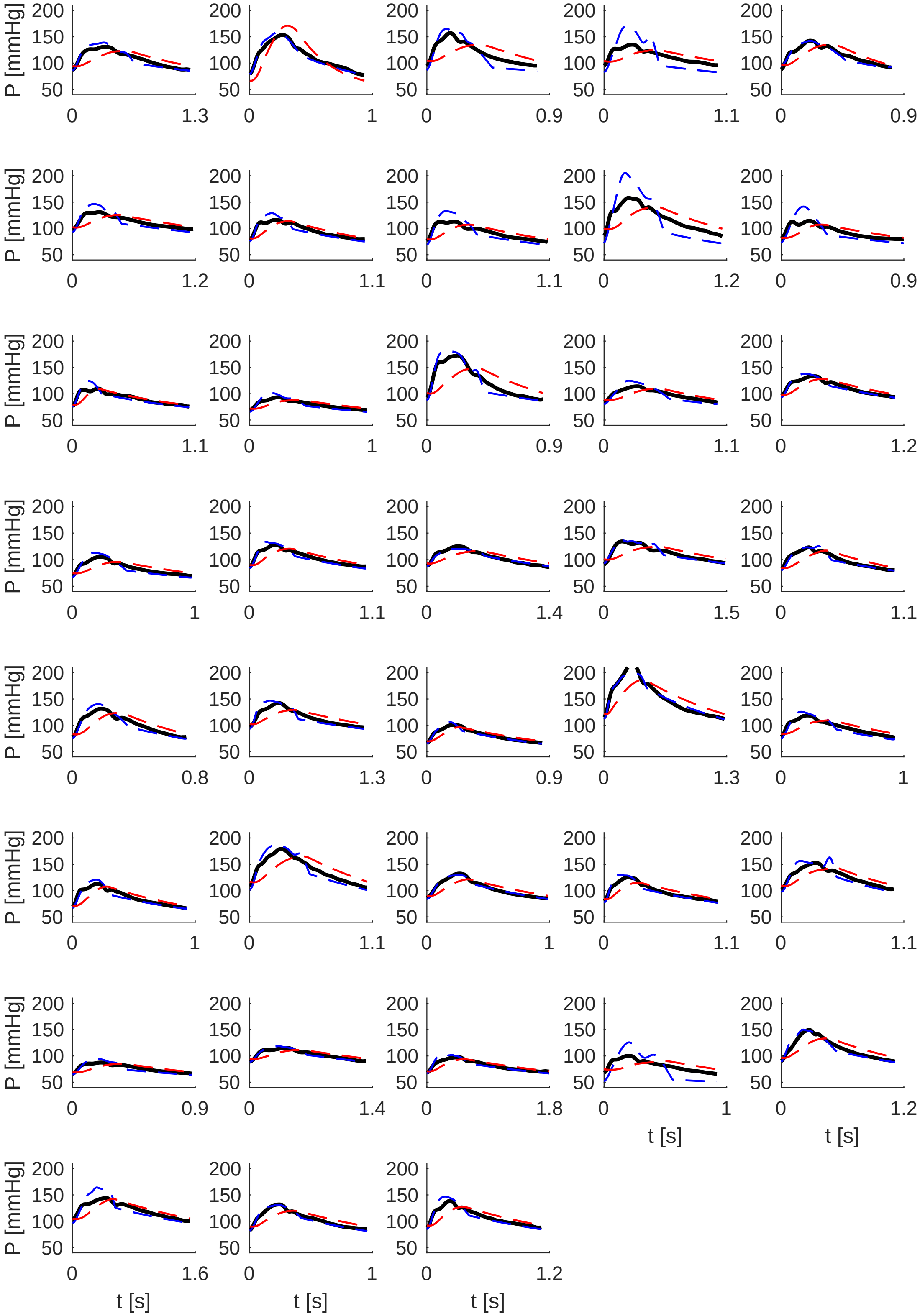

Figure S16: cBP wave estimations for ‘carotid-’. cBP waves calculated using the 2-element Windkessel (red lines) and 3-element Windkessel (blue lines) cBP algorithms. They are compared against reference cBP waves from the ‘Hypertensive’ dataset (thick black lines).
