# Supplementary material for: Estimating central blood pressure from aortic flow: development and assessment of algorithms
Source: Am J Physiol Heart Circ Physiol. Author manuscript; Available in PMC 2022 Mar 26. (PMC7612539; doi:10.1152/ajpheart.00241.2020)
Supplement: Appendix [file EMS143859-supplement-Appendix.pdf]

APPENDIX

APPENDIX A: DATASETS OF VIRTUAL SUBJECTS

Appendix A.1. 0-D Dataset: CV Parameter Variations

Appendix A.2. 1-D Dataset: Calculating Reference Z_0 and C_T Values at the Aortic Root

Reference Z_0 and C_T values for the 1-D dataset were calculated from aortic root BP (P) and flow (Q_{in}) waves using an in-house algorithm written in MATLAB and based on the 3-Wk model (Fig. A1). Assuming that P_{out} is known and that the total resistance $R_T = Z_0 + R$ is given by Eq. *1*, a parameter estimation problem can be solved for Z_0 and C_T. The estimated BP at time t_k can be written as

$$P(t_k) = f\left(Z_0', C_T', Q_{in}(t_k)\right) + e_k, \quad (A1)$$

with e_k the residual error between the estimated and reference BP at each time t_k, $k = 1, \ldots, K$, and Z_0' and C_T' the estimated parameters. The problem can be solved through iterative minimization of the cost function $\mathbf{e}^\top\mathbf{e}$, where $\mathbf{e}$ is the vector containing the residual errors at each time t_k. The iterative procedure starts from an initial estimate $\left(Z_{0,0}', C_{T,0}'\right)$. The parameters at iteration $i + 1$ are then calculated using the recursive equation

$$\left(Z_{0,i+1}', C_{T,i+1}'\right) = \left(Z_{0,i}', C_{T,i}'\right) - \mathbf{H}_i\mathbf{q}_i, \quad (A2)$$

where $\mathbf{H}_i$ and $\mathbf{q}_i$ are the Hessian and the gradient, respectively, of the cost function evaluated at iteration i. This equation can be obtained by approaching the cost function by a second-order Taylor expansion and minimizing the approached function. The mean cBP difference shown in Fig. A1, *B* and *C*, was calculated for each iteration as $\frac{1}{K}\sqrt{\sum_{k=1}^{K} e_k^2}$, with e_k the residual error at time t_k. The iterative procedure was stopped when either *1*) the change in both Z_0 and C_T estimates between iterations was smaller than 10^{-6} or *2*) after 15 iterations.

APPENDIX B: CARDIOVASCULAR PARAMETER ESTIMATION METHODS

All CV parameter estimation methods used in this study are described next. Novel methods are marked with an asterisk in the title.

Table A1. *CV parameter variations used for the three-element Windkessel (0-D) dataset*

	Variations					
	Negative		Baseline	Positive		
CV parameter, units	$\mu - \sigma$	$\mu - 0.5\sigma$	μ	$\mu + 0.5\sigma$	$\mu + \sigma$	References
SV, mL	71.2	79.8	88.4	97.0	105.7	(52)
HR, beats/min	52.9	60.8	68.8	76.7	84.7	(52)
P_{out}, mmHg	31.7	32.5	33.2	34.0	34.7	(51)
R_T, mmHg·s/mL	0.468	0.484	0.500	0.516	0.532	(44)
C_T, mL/mmHg	2.20	2.23	2.27	2.30	2.34	(50)
Z_0, mmHg·s/mL	0.0256	0.0358	0.0485	0.0644	0.0847	(65, 49)

μ and σ are mean and standard deviation values, respectively, for each CV parameter from the clinical literature. C_T: total arterial compliance; HR: heart rate; P_{out}: outflow vascular pressure; R_T: total arterial resistance; SV: stroke volume; Z_0: aortic characteristic impedance. These values are based on observations in healthy humans from the clinical literature.

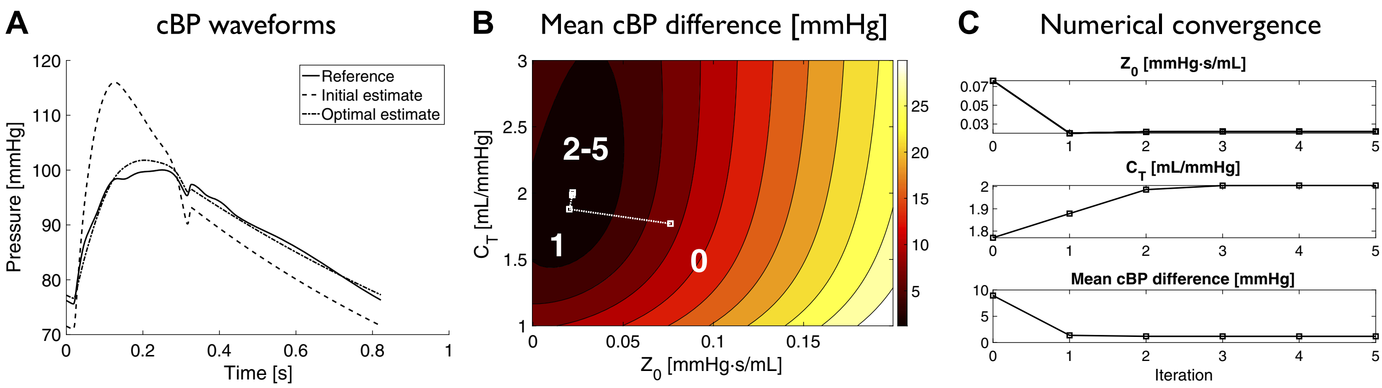

Figure A1. Extracting reference aortic characteristic impedance (Z_0) and total arterial compliance (C_T) values at the aortic root. *A:* reference central blood pressure (cBP) wave for a one-dimensional (1-D) model virtual subject, and corresponding initial and optimal estimates. *B*: contour plot (in mmHg) of the mean difference between the estimated and reference cBP waves, with Z_0 in the *x*-axis and C_T in the *y*-axis. Each iteration is shown in white squares; iterations 0 and 5 correspond to the initial and optimal cBP estimates, respectively. *C*: the values of Z_0, C_T, and the cBP mean difference are shown for the initial estimate and for every iteration until numerical convergence is reached.

Appendix B.1. LV—Left Ventricular Ejection Time, LVET

LV1—dP/dt analysis, 1.*

The method is described in the work by Charlton et al. (32). LVET corresponds to the point of peak pressure after the pressure systolic peak.

LV2—dP/dt analysis, 2.

This method is described in the work by Itu et al. (37). LVET coincides with the minimum of

$$\frac{\mathrm{d}P}{\mathrm{d}t}\left(0.5-\left|0.5-\frac{\mathrm{HR}\cdot t}{60}\right|\right)^2, \qquad (B1)$$

where P is a peripheral BP wave and HR represents the heart rate in beats/min.

LV3—0.37$\sqrt{T}$.

LVET is calculated using the empirical relationship described in the work by Bazett (31): $0.37\sqrt{T}$, where T is the duration of the cardiac cycle in seconds.

LV4—Q analysis.*

Q is analyzed from the global minimum after peak flow to 50% of T (Fig. B1). If all Q values are smaller than 1% of maximum Q, LVET corresponds to the time of the global minimum. Otherwise, starting from the time of the global minimum, all sign changes (from negative to positive), all maxima, and all zero values are found. LVET corresponds to either the first sign change, the first local maximum, or the first zero value (whichever one occurs first). If all else fails, method LV 3 is used.

Appendix B.2. OP—Outflow Pressure

OP1—diastolic decay fit, 1.

The concept of a diastolic decay fit was first described by Frank (15). P is analyzed between LVET and the end of diastole (P_d). The multidimensional unconstrained nonlinear minimization (Nelder–Mead) MATLAB function *fminsearch.m* is used to find the best fit between P_d and an exponential decay curve of the form: $P_{exp} = P_{out} + (P_{exp}(t_0) - P_{out})e^{-(t-t_0)/\tau}$, where t_0 = LVET. To avoid nonphysiological values of P_{out}, the following filters are applied: if $\tau < 0$ or $P_{out} < 0$, P_{out} is set to 0; and if $P_{out} \geq$ DBP, P_{out} is set to 0.5DBP.

OP2—diastolic decay fit, 2.

Similar to OP1, but using $t_0 = \frac{2}{3}\,\mathrm{LVET} + \frac{1}{3}T$ instead, as described by Simon et al. (44).

OP3—50% of DBP.*

P_{out} is estimated as 50% of DBP.

OP4—70% of DBP.

As suggested by Parragh et al. (56), P_{out} is estimated as 70% of DBP.

Appendix B.3. AR—Arterial Resistance

AR1—peripheral pressure waveform.

R_T is calculated using Eq. *1* and MBP is calculated as the mean of P.

AR2—peripheral DBP and SBP values.

Similarly to AR1, but using MBP = 0.4SBP + 0.6DBP instead, as described by Bos et al. (22).

Appendix B.4. AC—Arterial Compliance

AC1—2-point diastolic decay.

The concept of a diastolic decay fit was first described by Frank (15). Using only the first and last points of the diastolic part of P, C_T is calculated as:

$$\frac{T-\mathrm{LVET}}{\ln\left(\frac{P(\mathrm{LVET})-P_{out}}{\mathrm{DBP}-P_{out}}\right)R_T}. \qquad (B2)$$

AC2—diastolic decay fit, 1.

Given that $\tau = (R_T - Z_0)C_T$, OP1 can be used to calculate τ, and rearranging:

$$C_T = \frac{\tau}{R_T - Z_0}. \tag{B3}$$

If τ is negative, then P_{out} is set to 0 and τ is recalculated.

AC3—diastolic decay fit, 2.

Similar to AC2, but using $t_0 = \frac{2}{3}$ LVET $+ \frac{1}{3}T$ instead, as described by Simon et al. (44).

AC4—area method.

This method is described by Randall et al. (41). C_T is calculated as:

$$\frac{\int_{t_1}^{t_2}(P - P_{out})dt}{R_T(P(t_1) - P(t_2))}, \tag{B4}$$

where t_1 and t_2 are equal to $\frac{2}{3}$ LVET $+ \frac{1}{3}T$ and 90% of T, respectively.

AC5—two-area method.

This method is described by Self et al. (43). C_T is calculated by solving two simultaneous equations of the form:

$$\int_{t_1}^{t_2} Q dt - \frac{1}{R_T}\int_{t_1}^{t_2}(P - P_{out})dt = C_T(P(t_1) - P(t_2)), \tag{B5}$$

from the start of the cycle to LVET and from LVET to T.

AC6—diastolic blood pressure method*.

C_T is calculated by minimizing the relative error, DBP_{err} = $(DBP_{est} - DBP_{ref})/DBP_{ref}$, between the estimated (DBP_{est}) and reference (DBP_{ref}) values of DBP, as seen in Fig. B2. For each iteration, j, DBP_{est} is calculated as the minimum of the estimated BP, P_{est}, using the three-element Windkessel model (Eq. 5). The initial conditions are $C_{T,0}$ = SV/PP and $P_0 = DBP_{ref}$. While $DBP_{err} > 1\%$, $C_{T,j} = C_{T,j\text{-}1}/DBP_{err}^2$. C_T corresponds to the final value of $C_{T,j}$.

AC7—pulse pressure method.

This method is described by Stergiopulos et al. (25). Similar to AC6, but minimizing the relative PP error, PP_{err}, instead.

AC8—stroke volume over pulse pressure.

This method is described by Chemla et al. (27). C_T corresponds to SV/PP.

AC9—three-element Windkessel optimization*.

This method is described in appendix A.2. The initial value of C_T is calculated using AC8.

Appendix B.5. PV—Pulse Wave Velocity

The foot-to-foot (PV 1 and PV 2) and least-squares (PV 3 and PV 4) methods used here are described by Gaddum et al. (35). Both methods require the measurement of two pulse waves at both ends of a given arterial path of length L. The foot-to-foot method focuses on detecting the feet of both pulse waves to calculate the transit time (TT) between them. For each pulse wave, the foot is detected as the intersection between a horizontal projection of the minimum value and a projection of the maximum slope of the systolic upstroke.

The least-squares method calculates the sum of the squared differences between the systolic upstroke of both waves multiple times, by fixing one wave and shifting the other one by one datapoint at a time. The temporal shift that minimizes the squared differences is used to estimate TT. For both methods, PWV is then calculated as PWV = L/TT.

PV1—foot-to-foot: aortic flow.

The inputs are two noninvasive flow waves at the ascending and descending aortas.

PV2—foot-to-foot: carotid-femoral pressures.

The inputs are two noninvasive BP waves at the carotid and femoral arteries.

PV3—least-squares: aortic flow.

The inputs are two noninvasive flow waves at the ascending and descending aortas.

PV4—least-squares: carotid-femoral pressures.

The inputs are two noninvasive BP waves at the carotid and femoral arteries.

PV5—sum of squares.

This method has been adapted from the original one described by Davies et al. (34). PWV is calculated from the peripheral BP, P, and aortic flow, Q waves using

$$\text{PWV} = \frac{1}{\rho A}\sqrt{\frac{\sum dP^2}{\sum dQ^2}}, \tag{B6}$$

where ρ is the blood density, A is the cross-sectional area at the aortic root, dP and dQ are differences in P and Q, respectively, between two adjacent time points, and the sums extend over a cardiac cycle. P and Q do not need to be aligned in time.

Appendix B.6. Z—Aortic Characteristic Impedance

Method $Z2$ is sensitive to temporal misalignments between P and Q, so the following restrictions were applied to account for waves that were not recorded simultaneously and/or at the same site: *1)* P is shifted so that its value at the start of the cycle coincides with DBP, and *2)* Q is shifted so that its value at the start of the cycle is as close as possible to the intersection between the x-axis and the tangent of Q at the time of maximum dQ/dt in early systole.

Z1—frequency methods.

Frequency domain methods to estimate characteristic impedance (Z_0) are based on the Fourier analysis of P and Q extracted simultaneously at the ascending aorta. Z_0 is

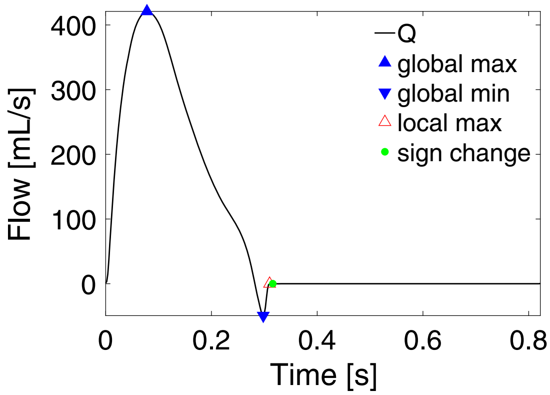

Figure B1. Novel method to estimate left ventricular ejection time (LVET) from the aortic flow wave, Q. LVET corresponds to the time of the first sign change (green circle), which occurs earlier than the local maximum (red triangle).

usually estimated as the average impedance modulus over a range of frequencies where fluctuations—due to wave reflections—above and below the characteristic impedance value are expected to cancel each other out. The following harmonic ranges, extracted from the literature, have been assessed in this study: 2–12th (40), 6–10th (42), 1–8th (33), 1–9th (23), 2–10th (38), 3–10th (36), 4–10th (45), 6–8th (29), and 4–8th (24) harmonics. These methods, in their original form, require P and Q measured simultaneously at the ascending aorta. However, for the proposed algorithms, a peripheral P measurement is used instead.

Z2—P-Q loop methods.

P-Q loop methods analyze the relationship between aortic P and Q during early systole, assuming that during this interval, the effects of wave reflections are minimal (23, 28), and hence

$$Z_0 \simeq \frac{P(t) - DBP}{Q(t) - Q(0)}, \qquad (B7)$$

where $Q(0)$ is the value of Q at the start of the cycle (normally zero). In this study, four P-Q loop methods were assessed where Z_0 was estimated as:

I the mean value of Eq. *B7* between the start of the cycle and the time of maximum Q;

II the slope of the linear least squares fit to the ratio between P and Q between the start of the cycle and the time of maximum flow;

III the value of Eq. *B7* at the time of maximum dQ/dt in early systole; and

IV the mean value of Eq. *B7* between the start of the cycle and the time of maximum dQ/dt in early systole.

The best-performing *P-Q* loop method, IV, was used to calculate the errors in Table 2. These methods, in their original form, require P and Q measured simultaneously at the ascending aorta. However, for the proposed algorithms, a peripheral P measurement is used instead.

Z3—5% of R_T.

As suggested by Murgo et al. (39), Z_0 is estimated as 5% of R_T.

Z4—approximated aortic characteristics.*

During early systole, wave reflections reaching the aortic root are assumed to be absent, and characteristic impedance can be estimated as $Z_0 = \Delta P/\Delta Q$, where ΔP and ΔQ are the changes in BP and flow rate, respectively (36). Peak flow, Q_{peak}, and the first systolic shoulder/peak, $P1$, occur at a similar time, so $\Delta Q = Q_{peak}$ and $\Delta P = P1$, and therefore, $Z_0 \simeq P1/Q_{peak}$, as seen in Fig. B3. Assuming that DBP and MBP remain constant within the large arteries, $P1$ is approximated as $MBP - DBP$ extracted from a peripheral P measurement. Hence, $Z_0 \simeq (MBP - DBP)/Q_{peak}$.

Z5—aortic characteristics.

This method is described by Westerhof et al. (47). Assuming that the aortic radius is much larger than the aortic wall thickness, Z_0 corresponds to $\rho PWV/A$, where ρ is the blood density, PWV is the aortic pulse wave velocity, and A is the aortic-root cross-sectional area.

Z6—three-element Windkessel optimization.*

This method is described in appendix A.2. The initial values of C_T and Z_0 are calculated using the AC8 and Z3 methods, respectively.

DATA ACCESS STATEMENT

A data supplement related to this manuscript is publicly available at https://doi.org/10.5281/zenodo.3968540. These materials are not a part of this manuscript and have not undergone peer review by the *American Physiological Society (APS)*. *APS* and the journal editors take no responsibility for these materials, for the website address, or for any links to or from it. The data collected during the literature review and the results from the 0-D and 1-D simulations, together with the MATLAB code used to generate 0-D datasets, to run 0-D simulations, to create input files for 1-D simulations, and to post-process and analyze these data are available here https://github.com/jmariscal-harana/cbp_estimation.

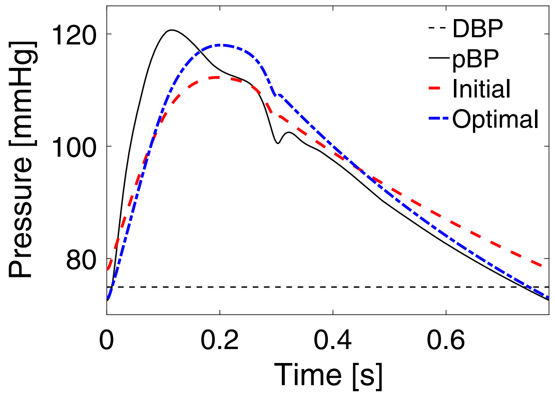

Figure B2. Novel iterative method to estimate arterial compliance (C_T) from the aortic flow and peripheral blood pressure (pBP) waves. C_T estimates are calculated by minimizing the relative error between the estimated and reference values of diastolic blood pressure (DBP). The latter is obtained from the pBP wave (black dashed line). The BP waves corresponding to the initial and optimal estimates of C_T are shown in red and blue lines, respectively.

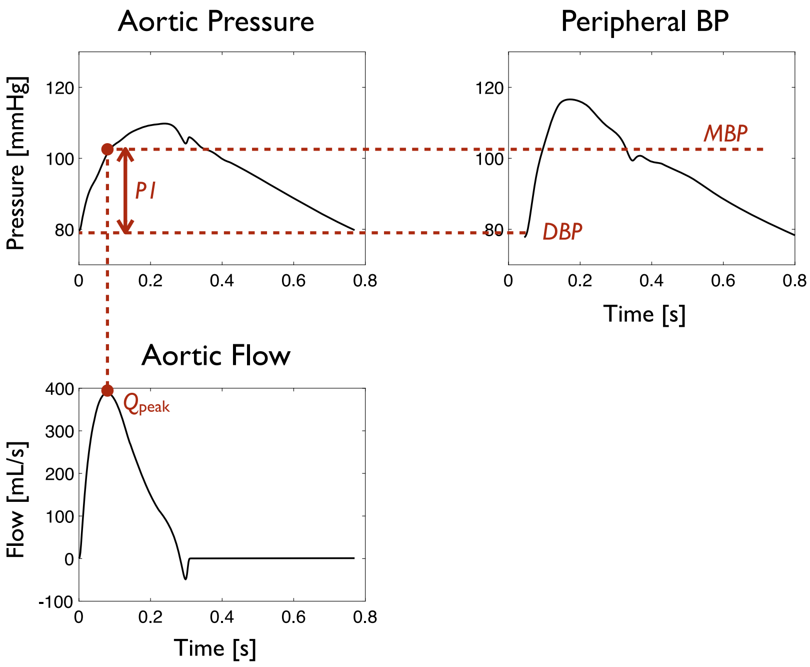

Figure B3. Novel method to estimate aortic characteristic impedance from the aortic flow and peripheral blood pressure (BP) waves. Pressure (*top*) and flow (*bottom*) waves at central (*left*) and peripheral (right) arterial locations for a subject from the 1-D dataset. The time of Q_{peak} and $P1$ is indicated by the vertical, red, dashed line. The value of $P1$ is approximated as mean blood pressure (MBP) − diastolic blood pressure (DBP) calculated from the peripheral BP wave.

Details of the code used to run the 1-D simulations are available at http://haemod.uk, and access requests should be addressed to J. Alastruey at jordi.alastruey-arimon@kcl.ac.uk. Details of how to replicate this study can be obtained by contacting J. Mariscal-Harana at jorge.mariscal_harana@kcl.ac.uk. Further information about the data and conditions of access can be found by emailing research.data@kcl.ac.uk.

GRANTS

This work was supported by a PhD Fellowship awarded by the King's College London and Imperial College London EPSRC Centre for Doctoral Training in Medical Imaging [EP/L015226/1], the British Heart Foundation (BHF) [PG/15/104/31913], and the Wellcome EPSRC Centre for Medical Engineering at King's College London [WT 203148/Z/16/Z]. The authors acknowledge financial support from the Department of Health through the National Institute for Health Research (NIHR) Cardiovascular MedTech Co-operative at Guy's and St Thomas' NHS Foundation Trust (GSTT). The views expressed are those of the authors and not necessarily those of the EPSRC, BHF, Wellcome Trust, NIHR, or GSTT.
